# Socioeconomic Inequalities in Receipt of Curative‐Intent Treatment and Survival After Surveillance‐Detected Hepatocellular Carcinoma: A Nationwide Cohort Study

**DOI:** 10.1111/apt.70753

**Published:** 2026-05-26

**Authors:** Hannes Hagström, Jeffrey V. Lazarus, Malin Sternby Eilard, Adam Bergman, Magnus Rizell, Per Sandström, Juan Vaz

**Affiliations:** ^1^ Department of Medicine, Huddinge Karolinska Institutet Stockholm Sweden; ^2^ Unit of Hepatology, Department of Upper GI Diseases Karolinska University Hospital Stockholm Sweden; ^3^ City University of New York Graduate School of Public Health & Health Policy (CUNY SPH) New York USA; ^4^ Barcelona Institute for Global Health (ISGlobal) Barcelona Spain; ^5^ Department of Transplantation Sahlgrenska University Hospital Gothenburg Region Västra Götaland Sweden; ^6^ School of Public Health and Community Medicine, Institute of Medicine, Sahlgrenska Academy University of Gothenburg Gothenburg Sweden; ^7^ Department of Biomedical and Clinical Sciences Linköping University Linköping Sweden; ^8^ Clinical Department of Surgery in Linköping Region Östergötland Linköping Sweden

**Keywords:** disparities, liver cancer, mortality, socioeconomic status, surveillance

## Abstract

**Background:**

Surveillance for hepatocellular carcinoma (HCC) aims to enable early diagnosis and receipt of curative‐intent treatment; however, socioeconomic status (SES) influences both participation in surveillance and subsequent outcomes. Whether SES‐related disparities persist among individuals diagnosed through surveillance remains unclear.

**Aims:**

To examine sociodemographic factors associated with receipt of curative‐intent treatment and mortality among individuals with surveillance‐detected HCC.

**Methods:**

We conducted a nationwide cohort study including adults with cirrhosis and surveillance‐detected HCC diagnosed in Sweden between January 1, 2012, and December 31, 2022. Sociodemographic data and outcomes were obtained through linkage to national registers. The primary outcome was receipt of curative‐intent treatment, analysed using multivariable logistic regression. Post‐treatment mortality was assessed using multivariable Cox regression.

**Results:**

Among 1,514 individuals with surveillance‐detected HCC, 845 (56%) were diagnosed at an early stage, and 974 (64%) received curative‐intent treatment. After adjustment for tumour characteristics, liver disease aetiology and severity, performance status, comorbidities, and calendar period, low SES was independently associated with a lower likelihood of receiving curative‐intent treatment (adjusted odds ratio 0.42, 95% CI 0.26–0.66, low vs. high SES). Among those treated with curative intent, low SES was also associated with higher mortality (adjusted hazard ratio 1.50, 95% CI 1.10–2.00).

**Conclusions:**

Socioeconomic inequalities in the receipt of curative‐intent treatment and survival persist even among individuals with surveillance‐detected HCC within a universal healthcare system. These findings indicate that surveillance alone is insufficient to ensure equitable outcomes and underscore the need for regionally tailored, people‐centred strategies that address social vulnerability across the HCC care continuum.

## Graphical abstract

Socioeconomic inequalities in receipt of curative‐intent treatment and survival persist among individuals with surveillance‐detected HCC within a universal healthcare system. These findings suggest that surveillance alone is insufficient to ensure equitable outcomes and highlight the need for strategies addressing social determinants across the care continuum.

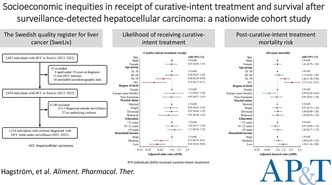

## 1 Introduction

Hepatocellular carcinoma (HCC) is a leading cause of cancer‐related mortality worldwide [1], with curative‐intent treatment largely restricted to individuals diagnosed at an early stage [2]. International guidelines, therefore, recommend surveillance for individuals living with cirrhosis who are potential candidates for treatment with curative intent, including selected individuals with decompensated cirrhosis who are eligible for liver transplantation [3, 4, 5]. Although surveillance improves early detection and access to curative‐intent treatment [6], uptake among individuals with cirrhosis remains limited; a meta‐analysis including 29 studies and 118,799 individuals from 10 countries reported a pooled surveillance utilisation of 24% [7].

Beyond tumour biology and liver disease severity [2, 8], sociodemographic factors influence HCC incidence, stage at diagnosis, treatment access, and survival [9]. In several European countries, including Sweden, lower socioeconomic status (SES) has been associated with higher HCC incidence and poorer outcomes [10, 11, 12]. However, it remains unclear whether such socioeconomic inequalities persist among individuals diagnosed through surveillance—an optimal point in the care pathway where early detection should, in principle, mitigate disparities. Although numerous studies have compared outcomes between surveillance‐detected and non‐surveillance‐detected HCC [6], no nationwide study has specifically examined sociodemographic factors and their associations with receipt of curative‐intent treatment or survival among individuals with surveillance‐detected HCC.

The primary aim of this study was to identify factors associated with failure to receive curative‐intent treatment among individuals diagnosed with HCC through surveillance in Sweden. Specifically, we examined whether sociodemographic characteristics were independently associated with receipt of curative‐intent treatment among surveillance‐detected HCC cases, and whether survival differed across these groups among individuals who received curative‐intent treatment. We hypothesised that socioeconomic disadvantage would be associated with a lower likelihood of receiving curative‐intent treatment and poorer survival, even among individuals diagnosed through surveillance.

## 2 Methods

### 2.1 Study Population

This study was based on data from the Swedish national quality register for cancers of the liver, gallbladder, and bile ducts (SweLiv). Established in 2008 and validated against the Swedish Cancer Register in 2014 [13], SweLiv currently captures more than 95% of all primary liver cancer cases nationwide [11].

We included all individuals aged ≥ 18 years who were registered in SweLiv with a diagnosis of HCC (International Classification of Diseases 10th Edition code C22.0) between January 1, 2012, and December 31, 2022. The start of the study period was chosen to coincide with the introduction of the first national guidelines for HCC management in Sweden [13], which were subsequently updated in 2015, 2020, and 2022, allowing evaluation of individuals diagnosed within a nationally standardised and evolving surveillance and treatment framework. The end of the study period was chosen because clinical data on received curative‐intent treatments were available through December 31, 2023, allowing for a minimum of one year of follow‐up after diagnosis for individuals diagnosed in 2022.

We excluded individuals who: (i) had missing or invalid sociodemographic information; (ii) had HCC not detected through surveillance, as defined by the diagnostic pathway variable in SweLiv (described below); or (iii) lacked underlying cirrhosis, as defined below. We restricted the study population to individuals with cirrhosis, as this group constitutes the primary target population for HCC surveillance [3, 4].

This study was approved by the Swedish Ethical Review Authority (reference nos. 2023–04555‐01 and 2024–05174‐02). The requirement for informed consent was waived because the study was based exclusively on retrospective analysis of registry data.

### 2.2 Sociodemographic Characteristics

Data on sociodemographic characteristics were obtained from Statistics Sweden. Sex was defined according to binary biological sex as recorded in national population registries. Region of birth was categorised into Nordic origin (Sweden, Norway, Denmark, Finland, Iceland, the Faroe Islands, Greenland, and Åland), other European origin, and non‐European origin. Marital status was classified as married (including cohabiting), single, divorced, or widowed.

SES was assessed both at the individual and contextual levels. Household income was used as the primary indicator of individual‐level SES, as it reflects current economic resources and has more complete coverage than educational data, which are more frequently missing among individuals born outside of Sweden [14]. Educational attainment was, nevertheless, included as a complementary SES measure and categorised as ≤ 9 years, 10–12 years, or > 12 years of schooling.

Household income was defined as equivalised disposable income per consumption unit, including all taxable and tax‐exempt income after deduction for taxes and negative transfers, and accounting for capital gains and losses [11, 14]. Income is reported to the Income and Taxation Register maintained by Statistics Sweden, based on administrative data reported to the Swedish Tax Agency, and is therefore not self‐reported. To allow comparisons across households of different sizes and compositions, Statistics Sweden adjusts disposable income using national consumption weights.

Household income was assessed for the year preceding HCC diagnosis, or if unavailable, for the second year prior. Individuals were categorised into income groups based on the national distribution of household income across all Swedish households: low (the lowest quartile), medium (second and third quartiles), and high (the highest quartile) [11]. This income‐based classification has previously been shown to be strongly associated with the risk of cirrhosis in the general Swedish population [15], as well as with HCC incidence and clinical outcomes in the overall HCC population [11, 16].

Residential area at the time of diagnosis and neighbourhood‐level socioeconomic data were obtained from Statistics Sweden using Demographic Statistical Areas (DeSO), small‐area units introduced to capture local socioeconomic conditions and segregation. During the study period, Sweden was divided into 5984 DeSOs, each comprising about 600–4,300 residents (median 1,600) [17]. For each calendar year, we derived the proportion of residents with low household income within each DeSO to reflect area‐level economic disadvantage. DeSOs were ranked annually according to this measure and categorised into national quintiles, ranging from least deprived (Q1) to most deprived (Q5) [11]. Each individual was assigned a neighbourhood deprivation category based on their residential DeSO at the time of HCC diagnosis. In addition, the degree of urbanisation (urban, semi‐urban, rural) was included to account for potential differences in healthcare access and geographical context.

### 2.3 Clinical Characteristics

Data on diagnostic pathways, cirrhosis status, underlying liver disease, tumour stage, and treatments were obtained primarily from SweLiv; if tumour stage was missing, this information was retrieved from the Cancer Register. As SweLiv has not yet been formally validated for cirrhosis status and liver disease aetiology, supplementary diagnostic information was retrieved from the National Patient Register (NPR), which has undergone extensive validation [18].

SweLiv records the diagnostic pathway for each individual at the time of registration. Individuals were classified as having surveillance‐detected HCC if the reporting clinician selected the corresponding predefined category. Diagnostic pathways are clinician‐reported and do not capture detailed information on surveillance modality, intensity, or adherence.

During the study period, Swedish HCC surveillance practice was aligned with recommendations from the European Association for the Study of the Liver, comprising semi‐annual ultrasonography for all eligible individuals with cirrhosis, irrespective of aetiology [3]. Surveillance is typically conducted within specialist hepatology care but may, in some regions, be coordinated by gastroenterology, infectious diseases, internal medicine, or primary care services. Data from primary care visits are not recorded in the NPR.

Underlying liver disease aetiology was determined using SweLiv and complemented by ICD‐10 codes from the NPR [11]. Six mutually exclusive categories were defined: hepatitis B (HBV), hepatitis C virus (HCV), alcohol‐related liver disease (ALD), metabolic dysfunction‐associated steatotic liver disease (MASLD), rare liver diseases (including autoimmune hepatitis, primary biliary cholangitis, and primary sclerosing cholangitis), and cryptogenic cirrhosis. Because some individuals may have more than one potential cause of cirrhosis, a hierarchical classification was applied to assign a primary underlying aetiology, prioritising, in order, HBV, HCV, ALD, rare liver diseases, and cryptogenic cirrhosis [11, 15].

Cirrhosis was identified using information from SweLiv and/or validated ICD‐10 codes in the NPR, which have been shown to have a positive predictive value exceeding 90% [19]. Cirrhosis status was thus defined using combined data sources, as previously described [11]. Liver disease severity was assessed using the Child–Pugh score reported in SweLiv at the time of HCC registration, with scores > 7 categorised as decompensated cirrhosis. Comorbid conditions, including type 2 diabetes, coronary artery disease, chronic kidney disease, chronic obstructive pulmonary disease, extrahepatic cancer diagnosed within 5 years prior to HCC diagnosis, alcohol use disorder, drug use disorder, and depression/anxiety, were identified using data from the NPR and the Prescribed Drug Register and included as covariates in multivariable analyses due to their relevance for HCC prognosis and/or treatment decision‐making [3, 4, 5].

### 2.4 Staging and Treatment

The Swedish treatment algorithm for HCC used during the study period was broadly based on the Barcelona Clinic Liver Cancer (BCLC) system [8], although the Swedish framework differs in several important aspects. Tumours are classified into four stages—early, intermediate, advanced, and terminal—corresponding to BCLC stages 0/A, B, C, and D, respectively [8]. In addition, performance status is assessed using all patient‐reported symptoms rather than being restricted to cancer‐related symptoms alone. As a result, in clinical practice, some patients with impaired liver function or an Eastern Cooperative Oncology Group (ECOG) performance status ≥ 2 may still be considered for curative options other than liver transplantation.

Treatment data were retrieved from SweLiv, and individuals were categorised according to whether they received treatment with curative intent or not during follow‐up. Curative‐intent treatment was defined as surgical resection, local ablation, or liver transplantation. All procedures with curative intention were cross‐checked against corresponding intervention codes in the NPR, and individuals with conflicting or unverifiable procedural data were excluded from further analyses.

All variables and definitions presented above and in preceding sections are described in further detail in the (Tables S1–S3).

### 2.5 Outcomes

The primary outcome was receipt of treatment with curative intent. A secondary outcome was mortality following the first curative‐intent treatment. All individuals were followed until death, emigration, or 24 May 2024, whichever occurred first.

### 2.6 Statistical Analysis

Continuous variables were summarised using medians and interquartile ranges (IQRs), and categorical variables were presented as counts and proportions. Missing data were reported as counts and proportions.

To identify factors associated with receipt of treatment with curative intent, we fitted multivariable logistic regression models and reported adjusted odds ratios (aORs) with corresponding 95% confidence intervals (CIs). Separate models for the individual and contextual level SES were fitted. The main model included age group, sex, region of birth, marital status, educational attainment, household income, cirrhosis aetiology, Child–Pugh score, largest tumour size, tumour number, benign portal vein thrombosis, ECOG performance status, comorbidities, and calendar period (2012–2015, 2016–2019, and 2020–2022). HCV was chosen as the reference category for aetiology, as it has previously been reported to be the most common underlying aetiology among individuals diagnosed with HCC while under surveillance in Sweden [20].

A separate model incorporated neighbourhood deprivation and degree of urbanisation as contextual SES indicators; in this model, region of birth, marital status, educational attainment, and household income were excluded, while all other covariates were retained. Extrahepatic metastases and tumour thrombosis were excluded from all models due to perfect correlation with non‐receipt of curative‐intent treatment.

Time from diagnosis to first curative‐intent treatment was analysed using quantile regression, stratified by treatment modality. Median differences were the primary focus, with additional analyses at the 75th and 90th percentiles to capture prolonged waiting times. Models were adjusted for the same sociodemographic and clinical covariates.

For survival analyses, follow‐up began at the date of first curative‐intent treatment, with time measured in years. Kaplan–Meier estimates with Greenwood 95% CIs were used to determine median survival and survival probabilities across sociodemographic groups. Mortality was analysed using Cox proportional hazards regression. Separate models were fitted for individual‐ and contextual‐level SES, mirroring the logistic regression approach. The main model included individual‐level sociodemographic factors (sex, region of birth, marital status, educational attainment, and household income) and was adjusted for age group, calendar period, cirrhosis aetiology, comorbidities, and treatment type (ablation, resection, or liver transplantation). Treatment type was included for confounding control and was not interpreted causally.

A corresponding model including neighbourhood deprivation and degree of urbanisation was fitted, excluding region of birth, marital status, educational attainment, and household income, while retaining all other covariates. Adjusted hazard ratios (aHRs) with 95% CIs were reported. The proportional hazards assumption was assessed using Schoenfeld residuals, and no evidence of violation was detected.

Several sensitivity analyses were conducted to assess the robustness of the main findings and the potential impact of treatment heterogeneity and waiting times (Data S1). Post‐treatment survival analyses were stratified by curative‐intent treatment modality, with separate Cox models fitted for ablation and resection; analyses for liver transplantation were not emphasised due to limited statistical power resulting from few observed events.

Additional analyses stratified individuals by time from diagnosis to treatment initiation (≤ 90 vs. > 90 days for ablation and resection), using clinically relevant thresholds to account for incomplete information on potential bridging or downstaging therapies. Furthermore, to evaluate the joint influence of individual‐ and contextual‐level SES, we performed sensitivity analyses including both sets of SES indicators simultaneously in the logistic and Cox regression models, as previously specified. All models were adjusted for the same sociodemographic and clinical covariates as in the primary analyses.

All analyses were performed using Stata v.19 (StataCorp, College Station, TX, USA).

## 3 Results

**FIGURE 1:**
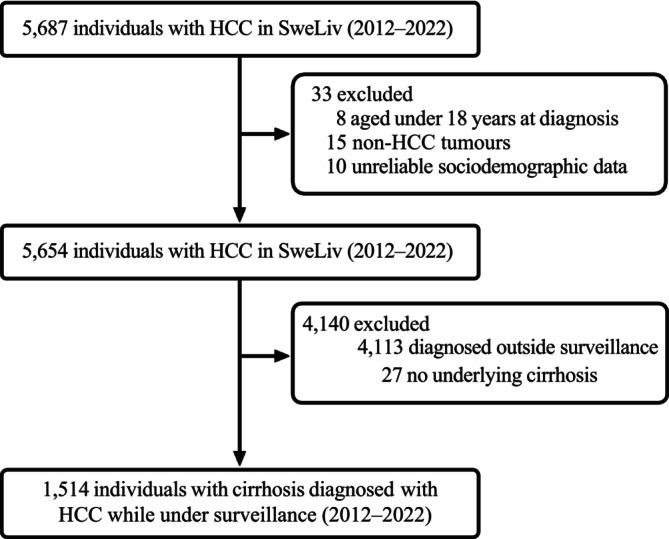
Flowchart of individuals aged ≥ 18 years with underlying cirrhosis and surveillance‐detected hepatocellular carcinoma (HCC) registered in the Swedish national quality register for cancers of the liver, gallbladder, and bile ducts (SweLiv), 2012–2022.

After exclusions, 1514 individuals remained (Figure 1). The median age at diagnosis was 66 years (IQR 60–73). Most individuals were male (*n* = 1,152; 76%) and born in a Nordic country (*n* = 1,240; 82%). Low household income was observed in 653 individuals (43%), and 484 (32%) had ≤ 9 years of formal education. Overall, 51% resided in the most deprived neighbourhoods (Q4–Q5), and the majority lived in urban areas (77%).

HCV was the most common underlying cause of HCC (40%), followed by ALD (24%) and HBV (12%). Compensated cirrhosis was present in 1313 individuals (87%), and 1209 (80%) had an ECOG performance status of 0–1. Sociodemographic and clinical characteristics stratified by receipt of curative‐intent treatment are summarised in Table 1. Clinical characteristics stratified by sex, region of birth, and household income are presented in the (Tables S4–S6).

**TABLE 1:** Sociodemographic and clinical characteristics of 1,514 individuals with surveillance‐detected hepatocellular carcinoma in sweden, 2012–2022.

		Curative‐intent treatment		Total
		Yes	No	
Individuals included		974 (64)	540 (36)	1,514 (100)
Sex	Male	738 (76)	414 (77)	1,152 (76)
	Female	236 (24)	126 (23)	362 (24)
Median age (IQR)		65 (60–72)	68 (61–74)	66 (60–73)
Age group	18–59	239 (25)	107 (20)	346 (23)
	60–69	410 (42)	191 (35)	601 (40)
	70–79	285 (29)	205 (38)	490 (32)
	80+	40 (4)	37 (7)	77 (5)
Region of birth	Nordic	783 (80)	457 (85)	1,240 (82)
	Europe (non‐Nordic)	76 (8)	33 (6)	109 (7)
	Non‐European	115 (12)	50 (9)	165 (11)
Marital status	Married	434 (45)	223 (41)	657 (43)
	Single	228 (23)	129 (24)	357 (24)
	Divorced	256 (26)	147 (27)	403 (27)
	Widowed	56 (6)	41 (8)	97 (6)
Educational	> 12 years	201 (21)	95 (18)	296 (20)
attainment	10–12 years	446 (46)	255 (47)	701 (46)
	≤ 9 years	305 (31)	179 (33)	484 (32)
	Unknown	22 (2)	11 (2)	33 (2)
Household	High	176 (18)	60 (11)	236 (16)
income	Medium	412 (42)	213 (39)	625 (41)
	Low	386 (40)	267 (50)	653 (43)
Neighbourhood	Q1 (least)	135 (14)	54 (10)	189 (12)
deprivation	Q2	151 (15)	92 (17)	243 (16)
	Q3	206 (21)	100 (19)	306 (20)
	Q4	192 (20)	121 (22)	313 (21)
	Q5 (most)	290 (30)	173 (32)	463 (31)
Degree of	Urban	759 (78)	411 (76)	1,170 (77)
urbanisation	Semi‐urban	77 (8)	38 (7)	115 (8)
	Rural	138 (14)	91 (17)	229 (15)
Period	2012–2015	293 (30)	166 (31)	459 (30)
	2016–2019	386 (40)	218 (40)	604 (40)
	2020–2022	295 (30)	156 (29)	451 (30)
Aetiology	HCV	395 (41)	214 (40)	609 (40)
	ALD	226 (23)	138 (25)	364 (24)
	HBV	134 (14)	49 (9)	183 (12)
	MASLD	100 (10)	60 (11)	160 (11)
	Rare liver diseases	86 (9)	46 (9)	132 (9)
	Cryptogenic	33 (3)	33 (6)	66 (4)
CP score	≤ 7	889 (91)	424 (79)	1,313 (87)
	> 7	85 (9)	116 (21)	201 (13)
ECOG PS	0–1	865 (89)	344 (64)	1,209 (80)
	≥ 2	109 (11)	196 (36)	305 (20)
Tumour size	Median (IQR)	22 (16–30)	40 (25–56)	26 (20–40)
largest	≤ 20 mm	438 (45)	84 (16)	522 (35)
	21–30 mm	304 (31)	106 (20)	410 (27)
	> 30 mm	232 (24)	350 (64)	582 (38)
*N* of tumours	1	653 (67)	234 (43)	887 (59)
	2–3	274 (28)	177 (33)	451 (30)
	> 3	44 (5)	122 (23)	166 (11)
	Uncertain	3 (< 1)	7 (1)	10 (< 1)
Thrombosis	Tumour	0	74 (14)	74 (5)
	Portal vein (benign)	30 (3)	101 (19)	131 (9)
Metastasis	Extrahepatic	0	46 (9)	46 (3)
Stage at	Early	759 (78)	86 (16)	845 (56)
diagnosis	Intermediate	215 (22)	176 (33)	391 (26)
	Advanced	0	104 (19)	104 (7)
	Terminal	0	174 (32)	174 (11)
Comorbidity	Type 2 diabetes	405 (42)	221 (41)	626 (41)
	CAD	117 (12)	69 (13)	186 (12)
	CKD	78 (8)	42 (8)	120 (8)
	COPD	104 (11)	90 (17)	194 (13)
	Extrahepatic cancer^a^	124 (13)	81 (15)	205 (14)
	Alcohol use disorder	202 (21)	117 (22)	319 (21)
	Drug use disorder	78 (8)	44 (8)	122 (8)
	Depression or anxiety	138 (14)	68 (13)	206 (14)

*Note:* Rare liver diseases included primary sclerosing cholangitis, primary biliary cholangitis, and autoimmune hepatitis.

Abbreviations: ALD, alcohol‐related liver disease; CAD, coronary artery disease; CKD, chronic kidney disease; COPD, chronic obstructive pulmonary disease; CP, Child‐Pugh; ECOG, Eastern Cooperative Oncology Group Performance Status; HBV, hepatitis B virus; HCV, hepatitis C virus; IQR, interquartile range; MASLD, metabolic dysfunction‐associated steatotic liver disease.

^a^ Extrahepatic cancers diagnosed within 5 years prior to hepatocellular carcinoma diagnosis.

Early‐stage HCC was identified in 845 individuals (56%), whereas 391 (26%) were diagnosed at an intermediate stage. Stage and treatment distribution stratified by sociodemographic characteristics is shown in Figure S1. A first curative‐intent treatment was received by 974 individuals (64%). Liver transplantation was the first curative‐intent treatment performed in 142 individuals (9%), surgical resection in 260 (17%), and ablation in 572 (38%).

### 3.1 Likelihood of Curative‐Intent Treatment Receipt

Logistic regression models were used to assess the association between sociodemographic and clinical characteristics and the likelihood of receiving curative‐intent treatment. As expected, clinical features included in the BCLC staging system, such as decompensated cirrhosis, ECOG performance status ≥ 2, increasing largest tumour size, and the presence of more than one tumour, were strongly associated with a lower likelihood of receiving curative‐intent treatment. Benign portal vein thrombosis was similarly associated with reduced odds of curative‐intent treatment (Table S7 and Table S8).

**FIGURE 2:**
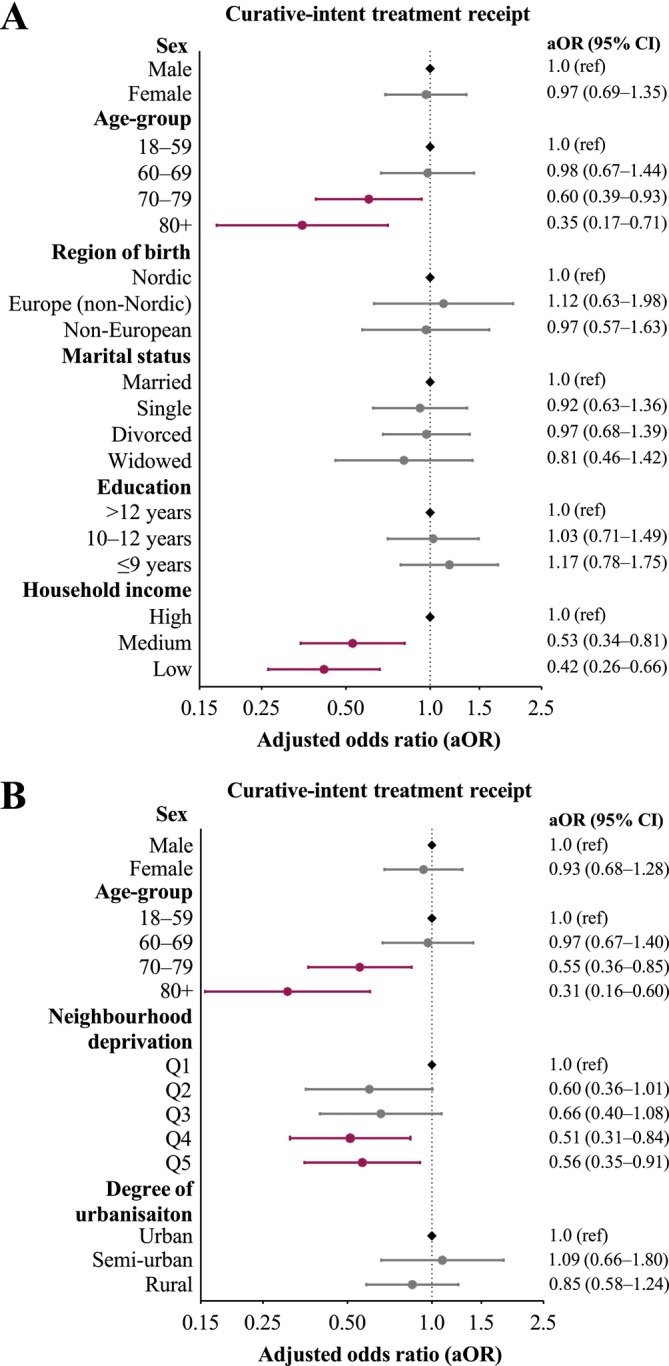
Adjusted odds ratios from multivariable logistic regression models for curative‐intent treatment receipt among individuals aged ≥ 18 years with surveillance‐detected hepatocellular carcinoma (HCC) in Sweden, 2012–2022. Panel A shows the model including individual‐level socioeconomic variables, and Panel B shows the model including contextual‐level socioeconomic variables. All models were adjusted for the variables shown, as well as cirrhosis aetiology, Child–Pugh score, largest tumour size, tumour number, benign portal vein thrombosis, ECOG performance status, comorbidities (type 2 diabetes, coronary artery disease, chronic kidney disease, chronic obstructive pulmonary disease, non‐HCC cancer diagnosed within 5 years before HCC diagnosis, alcohol use disorder, drug use disorder, and depression/anxiety), and calendar period (2012–2015, 2016–2019, and 2020–2022). CI: Confidence interval; ECOG: Eastern Cooperative Oncology Group.

In contrast, none of the examined comorbidities were statistically significantly associated with the likelihood of receiving curative‐intent treatment. Similarly, no associations were observed for sex, region of birth, marital status, educational attainment, or calendar period. However, individuals with medium or low household income had substantially lower odds of receiving curative‐intent treatment compared with those in the highest income group, with aORs of 0.53 (95% CI 0.34–0.81) and 0.42 (95% CI 0.26–0.66), respectively (Figure 2A). Compared to HCV, HBV was associated with higher odds of receiving curative‐intent treatment (aOR 2.26, 95% CI 1.35–3.77), whereas no statistically significant associations were observed for other aetiologies.

At the contextual level, residence in more deprived neighbourhoods (Q4–Q5) was associated with lower odds of receiving curative‐intent treatment compared with Q1 (Q4: aOR 0.51, 95% CI 0.31–0.84; Q5: aOR 0.56, 95% CI 0.35–0.91), while degree of urbanisation was not associated with treatment receipt (Figure 2B).

### 3.2 Time From Diagnosis to First Curative‐Intent Treatment

The median time from diagnosis to first curative‐intent treatment was 84 days (IQR 55–135), and was longest for liver transplantation (194 days, IQR 134–265), followed by resection (79 days, IQR 55–112) and ablation (77 days, IQR 50–119). Waiting times decreased markedly over calendar time, particularly for ablation with median reductions of 20 days (95% CI 5–34) in 2016–2019 and 41 days (95% CI 26–56) in 2020–2022, and for resection with a median reduction of 29 days (95% CI 11–46) in 2020–2022, while no consistent socioeconomic differences in time to treatment were observed. For transplantation, median waiting time decreased by approximately 80 days (95% CI 18–120), although waiting times remained more heterogeneous, likely reflecting clinical complexity rather than socioeconomic position.

### 3.3 Survival

During a total of 4481 person‐years of follow‐up among 974 individuals who received curative‐intent treatment, 438 deaths (45%) occurred. The median survival was 6.45 years (95% CI 5.80–7.29), with 1‐ and 5‐year survival probabilities of 0.94 (0.92–0.96) and 0.58 (0.55–0.61), respectively. When survival was assessed from the date of the first curative‐intent treatment, corresponding to 4104 person‐years of follow‐up, the median survival time was 5.98 years (95% CI 5.39–6.84). The corresponding 1‐ and 5‐year survival probabilities were 0.92 (0.90–0.94) and 0.56 (0.52–0.59), respectively.

Post curative‐intent treatment survival probabilities, as well as median survival stratified by sociodemographic factors, are presented in the (Table S9). Individuals with high household income had longer median survival compared with those with low income; however, this difference was not statistically significant (7.03 years, 95% CI 5.01–9.05 vs. 4.95 years, 95% CI 4.29–5.86).

Results from the multivariable Cox regression model are shown in Table 2. Increasing age was associated with higher mortality. Among sociodemographic factors, being born in a non‐Nordic European country was associated with lower mortality compared with being born in a Nordic country (aHR 0.59, 95% CI 0.38–0.92). Low household income was associated with higher mortality compared to high household income (aHR 1.50, 95% CI 1.10–2.00), whereas sex, marital status, and educational attainment were not associated with mortality.

**TABLE 2:** Multivariable estimates for post curative‐intent treatment mortality among 974 individuals with surveillance‐detected hepatocellular carcinoma in sweden, 2012–2022.

		Multivariable	
		aHR (95% CI)	*p*
Sex	Male	1.0 (ref)	—
	Female	1.01 (0.79–1.29)	0.957
Age group	18–59	1.0 (ref)	—
	60–69	1.13 (0.85–1.49)	0.407
	70–79	1.66 (1.20–2.30)	0.002
	80+	2.71 (1.61–4.57)	< 0.001
Region of birth	Nordic	1.0 (ref)	—
	Europe (non‐Nordic)	0.59 (0.38–0.92)	0.019
	Non‐European	0.95 (0.65–1.39)	0.796
Marital status	Married	1.0 (ref)	—
	Single	0.97 (0.73–1.30)	0.850
	Divorced	1.04 (0.80–1.36)	0.759
	Widowed	1.04 (0.70–1.54)	0.855
Educational	> 12 years	1.0 (ref)	—
attainment	10–12 years	0.87 (0.66–1.14)	0.314
	≤ 9 years	1.02 (0.77–1.37)	0.876
Household income	High	1.0 (ref)	—
	Medium	1.08 (0.82–1.43)	0.590
	Low	1.50 (1.10–2.00)	0.010
Period	2012–2015	1.0 (ref)	—
	2016–2019	0.77 (0.61–0.97)	0.024
	2020–2022	0.78 (0.57–1.06)	0.113
Aetiology	HCV	1.0 (ref)	—
	ALD	1.56 (1.18–2.05)	0.002
	HBV	1.07 (0.75–1.53)	0.694
	MASLD	1.24 (0.86–1.78)	0.256
	Rare liver diseases	1.06 (0.71–1.62)	0.768
	Cryptogenic	1.66 (0.95–2.91)	0.074
Comorbidity	Type 2 diabetes	1.07 (0.86–1.33)	0.540
	CAD	1.16 (0.87–1.53)	0.297
	CKD	1.59 (1.14–2.22)	0.007
	COPD	1.45 (1.09–1.93)	0.011
	Extrahepatic cancer^a^	1.29 (0.99–1.68)	0.059
	Alcohol use disorder	1.07 (0.92–1.25)	0.367
	Drug use disorder	0.82 (0.64–1.05)	0.100
	Depression/anxiety	0.90 (0.74–1.06)	0.103
Treatment	Transplantation	1.0 (ref)	—
	Resection	2.91 (1.91–4.45)	< 0.001
	Ablation	3.62 (2.42–5.41)	< 0.001

*Note:* Results from a multivariable Cox regression model including all the variables in this table. Each individual was followed up from the date of the first curative‐intent treatment (transplantation, resection, or ablation) until the date of death, emigration, or 24 May 2024, whichever occurred first. Rare liver diseases included primary sclerosing cholangitis, primary biliary cholangitis, and autoimmune hepatitis.

Abbreviations: ALD, alcohol‐related liver disease; CAD, coronary artery disease; CI, confidence interval; CKD, chronic kidney disease; COPD, chronic obstructive pulmonary disease; HBV, hepatitis B virus; HCV, hepatitis C virus; HR, hazard ratio; MASLD, metabolic dysfunction‐associated steatotic liver disease.

^a^ Extrahepatic cancers diagnosed within 5 years prior to hepatocellular carcinoma diagnosis.

ALD was associated with higher mortality compared with HCV (aHR 1.47, 95% CI 1.11–1.94). Among comorbidities, chronic kidney disease and chronic obstructive pulmonary disease were associated with increased mortality (aHR 1.59, 95% CI 1.14–2.22 and aHR 1.45, 95% CI 1.09–1.93, respectively), whereas none of the other examined comorbidities were associated with mortality. Calendar period was associated with improved survival, with lower mortality observed for individuals diagnosed during 2016–2019 compared with 2012–2015 (aHR 0.77, 95% CI 0.61–0.97), whereas no statistically significant associations were observed for 2020–2022. Treatment modality was included for adjustment only; compared with liver transplantation, ablation and resection were associated with higher mortality, reflecting underlying differences in disease severity and treatment selection rather than causal treatment effects.

At the contextual level, neither neighbourhood deprivation nor degree of urbanisation was associated with mortality (Table S10).

### 3.4 Sensitivity Analyses

Results from sensitivity analyses addressing treatment heterogeneity and treatment timing were consistent with the primary findings (Table S11 and Table S12). When survival analyses were stratified by curative‐intent treatment modality, the direction of associations for key sociodemographic variables was broadly similar across ablation and resection, although several estimates were imprecise due to limited sample size within strata.

Socioeconomic differences in survival were most evident among individuals treated with ablation, where low household income remained associated with higher mortality (aHR 1.51, 95% CI 1.03–2.21). Stratification by time from diagnosis to treatment initiation yielded comparable results. For ablation and resection, analyses stratified at 90 days showed no material differences in sociodemographic associations between individuals treated earlier vs. later (data not shown).

In models including individual‐ and contextual‐level SES indicators simultaneously, estimates for household income were similar compared with the main model, whereas associations between neighbourhood deprivation and treatment receipt were attenuated and no longer statistically significant after adjustment for household income (Table S13 and Table S14).

## 4 Discussion

In this nationwide study of 1514 individuals with surveillance‐detected HCC in Sweden, more than half were diagnosed at an early stage, and nearly two‐thirds received curative‐intent treatment. Despite these favourable overall indicators, socioeconomic inequities in outcomes persisted. Household income and neighbourhood deprivation were independently associated with receipt of curative treatment, with individuals in the highest income group, or living in the least deprived neighbourhoods, having a higher likelihood of treatment compared with those in the low‐income group and those residing in more deprived neighbourhoods (Q4–Q5), even after comprehensive adjustment for tumour characteristics, liver disease severity, performance status, and comorbidities. These disparities were not limited to treatment allocation but translated into higher post‐curative‐intent treatment mortality among individuals with low household income.

Sweden represents a critical test case for equity in HCC care [11, 16]. The implementation of structured surveillance has improved early detection, increased the use of curative‐intent treatment, and contributed to improved survival at the population level [11, 20, 21, 22]. Within a setting of universal health care and structured HCC surveillance, our findings indicate that socioeconomic inequities persist even after diagnosis within a surveillance programme, where access to care would be expected to be more standardised. This suggests that factors beyond tumour stage and liver disease severity influence treatment allocation and outcomes.

At the individual level, household income has been shown to be a particularly salient marker of disadvantage in HCC [11, 16, 20]. Unlike education or marital status, income may more directly capture material constraints, competing life demands, and the capacity to engage with complex, multidisciplinary cancer care. For individuals living with chronic liver disease, often characterised by multimorbidity, psychosocial stressors, and fragmented care, these factors may critically influence attendance at appointments, adherence to recommendations, and navigation across transitions from surveillance to diagnosis and treatment [23].

At the contextual level, residence in more deprived neighbourhoods was associated with lower odds of receiving curative‐intent treatment, although this association was attenuated when household income was included simultaneously, indicating partial overlap between individual and area‐level disadvantage.

The mechanisms underlying these associations cannot be fully determined from register data. Still, the persistence of income‐related differences after adjustment for detailed clinical characteristics suggests that non‐clinical factors influencing access to and delivery of care may play a role, even within a universal healthcare system. In many settings, surveillance is largely confined to specialist care, creating structural barriers for individuals with lower health literacy or limited ability to navigate complex referral pathways [23, 24]. However, the lack of association with the degree of urbanisation argues against geographic access alone as an explanation for these differences.

Importantly, no consistent socioeconomic differences were observed in time from diagnosis to treatment, and sensitivity analyses accounting for treatment modality and timing yielded similar results. Together, these findings suggest that the observed disparities are unlikely to be explained solely by delays in care or treatment sequencing.

The association between comorbidities and post‐treatment mortality further highlights the need for integrated, person‐centred care. Chronic kidney disease and chronic obstructive pulmonary disease were associated with higher mortality risk, suggesting that outcomes after curative‐intent treatment are shaped not only by tumour control but by the broader health context in which individuals live. This underscores the importance of coordinated management of comorbidities and tailored follow‐up, particularly for individuals undergoing less invasive curative‐intent treatments [25].

The lower mortality risk observed among individuals born in non‐Nordic European countries may reflect selection effects, such as a healthy migrant effect or differences in underlying risk profiles, rather than a protective effect of country of birth. Residual confounding related to disease severity or functional status, which cannot be fully captured in registry data, may also contribute.

Although survival improved during the study period, likely reflecting advances in HCC management, the persistence of income‐related disparities indicates that clinical progress alone does not ensure equity. Without explicit attention to social determinants of health, innovations risk preferentially benefiting those already best positioned to access and navigate care.

Finally, our findings have clear implications for policy and clinical practice. Equity should be treated as a core quality indicator in HCC surveillance and treatment programmes, with routine monitoring of treatment allocation and outcomes by SES [9]. People‐centred interventions, such as health‐literacy‐sensitive communication, and structured follow‐up after surveillance detection, should be embedded within HCC care pathways to support individuals at higher risk of disadvantage [23, 24, 26, 27, 28].

### 4.1 Strength and Limitations

A major strength of this study is the use of nationwide, population‐based register data capturing more than 95% of all individuals diagnosed with HCC in Sweden, minimising selection bias and enhancing generalisability. The linkage of high‐quality clinical data from SweLiv with comprehensive national sociodemographic and health registers enabled detailed adjustments, providing coverage of key variables underpinning the BCLC staging system [8], with minimal missing data. Restricting the study population to individuals with surveillance‐detected HCC reduced heterogeneity in diagnostic pathways and allowed a focused assessment of equity within a structured surveillance context. In addition, analysing survival from the date of curative‐intent treatment enabled evaluation of post‐treatment outcomes while minimising the risk of immortal time bias. To our knowledge, this is the first nationwide study to assess associations between SES, receipt of curative‐intent treatment, and post‐treatment survival among individuals with surveillance‐detected HCC.

Several limitations should be considered when interpreting these findings. First, although restricting the cohort to individuals with surveillance‐detected HCC reduced heterogeneity in diagnostic pathways and minimised lead‐time bias, detailed information on surveillance modality, intensity, and adherence was not available. Second, information on bridging or downstaging treatments prior to ablation, resection, or transplantation was not available. Consequently, some individuals with longer waiting times may have received interim locoregional or systemic therapy, which could not be fully accounted for in the analyses.

Third, SES was captured using register‐based indicators. While we incorporated both individual‐level measures (household income and educational attainment) and contextual‐level measures (neighbourhood deprivation and degree of urbanisation), these proxies may not fully capture all dimensions of SES, such as wealth, health literacy, social support, or trust in healthcare. Household income, although derived from high‐quality administrative data and not self‐reported, does not fully reflect assets such as savings or home ownership, although several components of wealth may be indirectly captured through taxation. In addition, income measured in the year preceding diagnosis may be influenced by pre‐existing ill health.

Fourth, despite adjustment for a broad range of clinical characteristics and comorbidities, residual confounding related to disease severity or functional status cannot be entirely excluded. Fifth, as Swedish registers do not capture ethnicity or gender identity, we were unable to explore potential inequities related to these dimensions, which may be relevant in the context of liver disease and cancer outcomes.

Importantly, to address several of these limitations, we conducted multiple sensitivity analyses, including stratification by curative‐intent treatment modality and by waiting time to treatment initiation. The consistency of results across these analyses supports the robustness of the main findings and suggests that the observed sociodemographic associations were not primarily driven by treatment heterogeneity or differences in waiting time.

## 5 Conclusion

In this nationwide study conducted within Sweden's universal healthcare system, socioeconomic inequalities persisted in both the likelihood of receiving curative‐intent treatment and post‐treatment mortality risk among individuals with surveillance‐detected HCC. These findings demonstrate that surveillance alone is insufficient to ensure equitable outcomes and underscore the need for equity‐oriented approaches that address social vulnerability across the HCC care continuum.

## Author Contributions

**Hannes Hagström:** conceptualization, methodology, investigation, funding acquisition, writing – original draft. **Jeffrey V. Lazarus:** writing – review and editing. **Malin Sternby Eilard:** conceptualization, writing – review and editing. **Adam Bergman:** conceptualization, writing – review and editing. **Magnus Rizell:** conceptualization, writing – review and editing. **Per Sandström:** conceptualization, writing – review and editing. **Juan Vaz:** conceptualization, methodology, data curation, formal analysis, investigation, visualization, supervision, funding acquisition, writing – original draft, project administration.

## Funding

This work was supported by the Swedish Society of Medicine; the Swedish Foundation for Transplant and Cancer Research; the Swedish Gastroenterology Fund (Mag‐Tarmfonden); Tore Nilsons Stiftelse för Medicinsk Forskning; Åke Wiberg Stiftelse; Magnus Bergvalls Stiftelse; and Stiftelsen Lars Hiertas Minne.

## Conflicts of Interest

HH's institutions have received research funding from AstraZeneca, Echosens, Gilead, Intercept, MSD, Novo Nordisk, Takeda, and Pfizer. He has served as a consultant, speaker, or on advisory boards for AstraZeneca, Boehringer Ingelheim, Bristol Myers‐Squibb, GSK, Echosens, Ipsen, MSD, and Novo Nordisk and has been part of hepatic events adjudication committees for Arrowhead, Boehringer Ingelheim, KOWA, and GW Pharma. This has all been outside of this work. J.V.L. has received grants to his institutions from Boehringer Ingelheim, Echosens, Gilead Sciences, Madrigal Pharmaceuticals, MSD, Novo Nordisk, and Pfizer, consulting fees from Echosens, GSK, Madrigal Pharmaceuticals, Novo Nordisk, Pfizer, and Sonic Incytes, and honoraria for lectures from Echosens, GSK, Novo Nordisk, and Pfizer outside of this work. J.V. has received consulting fees from Roche and AstraZeneca and a research grant from Eisai outside of this work. J.V. has also received grants for this study from The Swedish Society of Medicine, The Swedish Foundation for Transplant and Cancer Research, and The Swedish Gastroenterology Fund, which partially financed this study. M.S.E., AB, MR, and P.S. have no conflicts of interest to declare.

## Supporting information


**Data S1:** Supporting methods.
**Table S1:** Sociodemographic variables.
**Table S2:** Clinical variables: Liver diseases and comorbidity.
**Table S3:** Clinical variables: Tumour‐related, performance status, and treatment.
**Table S4:** Sociodemographic and clinical characteristics of 1514 individuals with surveillance‐detected hepatocellular carcinoma in Sweden, 2012–2022, stratified by sex.
**Table S5:** Sociodemographic and clinical characteristics of 1,514 individuals with surveillance‐detected hepatocellular carcinoma in Sweden, 2012–2022, stratified by region of birth.
**Table S6:** Sociodemographic and clinical characteristics of 1,514 individuals with surveillance‐detected hepatocellular carcinoma in Sweden, 2012–2022, stratified by household income.
**Table S7:** Factors associated with the likelihood of curative‐intent treatment receipt among 1,514 individuals with surveillance‐detected hepatocellular carcinoma in Sweden, 2012–2022 (individual‐level socioeconomic status).
**Table S8:** Factors associated with the likelihood of curative‐intent treatment receipt among 1,514 individuals with surveillance‐detected hepatocellular carcinoma in Sweden, 2012–2022 (contextual‐level socioeconomic status).
**Table S9:** Post curative‐intent treatment survival probabilities and median survival among 974 individuals with surveillance‐detected hepatocellular carcinoma in Sweden, 2012–2022.
**Table S10:** Multivariable estimates for post curative‐intent treatment mortality among 974 individuals with surveillance‐detected hepatocellular carcinoma in Sweden, 2012–2022 (contextual‐level socioeconomic status).
**Table S11:** Multivariable estimates for post‐ablation mortality among 572 individuals with surveillance‐detected hepatocellular carcinoma in Sweden, 2012–2022.
**Table S12:** Multivariable estimates for post‐resection mortality among 260 individuals with surveillance‐detected hepatocellular carcinoma in Sweden, 2012–2022.
**Table S13:** Factors associated with the likelihood of curative‐intent treatment receipt among 1,514 individuals with surveillance‐detected hepatocellular carcinoma in Sweden, 2012–2022 (individual‐ and contextual‐level socioeconomic status).
**Table S14:** Multivariable estimates for post curative‐intent treatment mortality among 974 individuals with surveillance‐detected hepatocellular carcinoma in Sweden, 2012–2022 (individual‐ and contextual‐level socioeconomic status).
**Figure S1:** Proportions of stage at diagnosis and received treatment, stratified by sociodemographic characteristics and aetiology, among individuals aged ≥ 18 years with surveillance‐detected hepatocellular carcinoma (HCC) in Sweden, 2012–2022.

## Data Availability

Datasets generated and analysed during the current study are not publicly available due to legal restrictions, but additional analyses may be requested from the corresponding author upon reasonable request. External researchers may request the raw data from Swedish healthcare registers and perform additional analyses.
