# Supplementary material for: Socioeconomic Inequalities in Receipt of Curative‐Intent Treatment and Survival After Surveillance‐Detected Hepatocellular Carcinoma: A Nationwide Cohort Study
Source: Aliment Pharmacol Ther. 2026 May 26;64(5):647–58. doi: 10.1111/apt.70753 (PMC13466149; doi:10.1111/apt.70753)
Supplement: Supplementary file 1 [file APT-64-647-s001.docx]

**Socioeconomic inequalities in access to curative-intent treatment and survival after surveillance-detected hepatocellular carcinoma: a nationwide cohort study**

Hannes Hagström, Jeffrey V Lazarus, Malin Sternby Eilard, Adam Bergman, Magnus Rizell, Per Sandström, Juan Vaz

Supporting information

**Table of contents**

[Supporting methods	3](#_Toc228294178)

[Table S1. Sociodemographic variables	4](#_Toc228294179)

[Table S2. Clinical variables: liver diseases and comorbidity	5](#_Toc228294180)

[Table S3. Clinical variables: tumour related, performance status, and treatment	6](#_Toc228294181)

[Table S4. Sociodemographic and clinical characteristics of 1514 individuals with surveillance-detected hepatocellular carcinoma in Sweden, 2012–2022, stratified by sex	7](#_Toc228294182)

[Table S5. Sociodemographic and clinical characteristics of 1514 individuals with surveillance-detected hepatocellular carcinoma in Sweden, 2012–2022, stratified by region of birth	9](#_Toc228294183)

[Table S6. Sociodemographic and clinical characteristics of 1514 individuals with surveillance-detected hepatocellular carcinoma in Sweden, 2012–2022, stratified by household income	11](#_Toc228294184)

[Table S7. Factors associated with the likelihood of curative-intent treatment receipt among 1514 individuals with surveillance-detected hepatocellular carcinoma in Sweden, 2012–2022 (individual-level socioeconomic status)	13](#_Toc228294185)

[Table S8. Factors associated with the likelihood of curative-intent treatment receipt among 1514 individuals with surveillance-detected hepatocellular carcinoma in Sweden, 2012–2022 (contextual-level socioeconomic status)	14](#_Toc228294186)

[Table S9. Post curative-intent treatment survival probabilities and median survival among 974 individuals with surveillance-detected hepatocellular carcinoma in Sweden, 2012–2022	15](#_Toc228294187)

[Table S10. Multivariable estimates for post curative-intent treatment mortality among 974 individuals with surveillance-detected hepatocellular carcinoma in Sweden, 2012–2022 (contextual-level socioeconomic status)	16](#_Toc228294188)

[Table S11. Multivariable estimates for post-ablation mortality among 572 individuals with surveillance-detected hepatocellular carcinoma in Sweden, 2012–2022	17](#_Toc228294189)

[Table S12. Multivariable estimates for post-resection mortality among 260 individuals with surveillance-detected hepatocellular carcinoma in Sweden, 2012–2022	18](#_Toc228294190)

[Table S13. Factors associated with the likelihood of curative-intent treatment receipt among 1514 individuals with surveillance-detected hepatocellular carcinoma in Sweden, 2012–2022 (individual- and contextual-level socioeconomic status)	19](#_Toc228294191)

[Table S14. Multivariable estimates for post curative-intent treatment mortality among 974 individuals with surveillance-detected hepatocellular carcinoma in Sweden, 2012–2022 (individual- and contextual-level socioeconomic status)	20](#_Toc228294192)

# **Supporting methods**

Among individuals who received curative-intent treatment, time from diagnosis to curative-intent treatment initiation was analysed using quantile regression. Time to treatment was defined as the number of days between the date of diagnosis and the date of first curative-intent treatment. Analyses were performed separately for each treatment modality (ablation, resection, and liver transplantation) to account for expected differences in waiting times across treatment types.

Quantile regression was used to estimate differences in waiting time at specified quantiles of the distribution, with primary focus on the median (50th percentile). Additional analyses were conducted at higher percentiles (75th and 90th) to assess sociodemographic differences among individuals experiencing the longest waiting times. This approach was chosen because waiting times were right-skewed and because quantile regression does not rely on distributional assumptions, allowing robust estimation across the waiting time distribution.

Sociodemographic variables, including sex, age group, region of birth, marital status, educational attainment, and household income were included as the main exposures of interest. Models were adjusted for cirrhosis aetiology, Child–Pugh score, performance status, calendar period, and comorbidities. Regression coefficients from quantile regression were interpreted as absolute differences in waiting time (days) between groups at the specified quantiles.

To evaluate the robustness of the main findings and to assess the potential impact of treatment timing and treatment heterogeneity, several sensitivity analyses were conducted.

First, post-treatment survival analyses were stratified by curative-intent treatment modality. Separate Cox proportional hazards models were fitted within each treatment stratum to examine whether sociodemographic associations with survival were consistent across treatment types and to minimise residual confounding related to differences in prognosis between treatment modalities. Owing to the low number of observed and expected deaths among liver transplant recipients, stratified survival analyses for transplantation were considered underpowered and were therefore not emphasised in the interpretation. Sensitivity analyses focusing on treatment heterogeneity were consequently restricted to individuals treated with ablation or resection.

Second, to explore whether prolonged waiting times influenced the observed associations, additional sensitivity analyses were performed stratifying individuals according to time from diagnosis to treatment initiation. For individuals undergoing ablation or resection, analyses were stratified using a threshold of 90 days, with individuals categorised as treated within or beyond this interval. For liver transplantation, a 180-day threshold was explored descriptively, reflecting the longer and more variable waiting times typically observed for transplantation, but results were interpreted cautiously due to limited statistical power.

These waiting-time thresholds were chosen to account for incomplete information on locoregional palliative or bridging therapies, such as TACE, for which reliable treatment dates were unavailable. Because we could not exclude that individuals experiencing longer waiting times may have received unrecorded bridging or downstaging treatments prior to curative-intent therapy, stratification by clinically relevant waiting-time intervals was used to assess the robustness of the primary findings to potential unmeasured treatment complexity.

Within each stratum, post-treatment survival was analysed using Cox proportional hazards regression, adjusting for the same set of sociodemographic and clinical covariates as in the primary analyses. These sensitivity analyses were conducted to evaluate whether socioeconomic differences in post-treatment survival differed between individuals treated earlier versus later and to further assess the robustness of the main results.

| Table S1. Sociodemographic variables | | |
| --- | --- | --- |
| Variable | **Definition** | **Source** |
| Sex | Male or Female (biological) | Statistics Sweden |
| Age | Age at hepatocellular carcinoma diagnosis | Statistics Sweden |
| Region of birth | Nordic: Sweden, Denmark, Finland, Norway, Iceland, the Faroe Island, Greenland, and Åland | Statistics Sweden |
|  | European (non-Nordic): all other European countries |  |
|  | Non-European: all other countries |  |
| Marital status | Married: legally married or cohabiting | Statistics Sweden |
|  | Single |  |
|  | Divorced: legally divorced or separated |  |
|  | Widowed |  |
| Educational | > 12 years of formal education | Statistics Sweden |
| attainment | 10–12 years of formal education |  |
|  | ≤9 years of formal education |  |
| Household income^a^ | Based on the distribution of households in Sweden the year prior to hepatocellular carcinoma diagnosis | Statistics Sweden |
|  | High: highest quartile, >75% (wealthiest) |  |
|  | Medium: second and third quartiles, 25-75% |  |
|  | Low: lowest quartile, <25% (poorest) |  |
| Neighbourhood | Q1 (least) | Statistics Sweden |
| deprivation^b^ | Q2 |  |
|  | Q3 |  |
|  | Q4 |  |
|  | Q5 (most) |  |
| Degree of | Urban: areas located within the municipal centre | Statistics Sweden |
| urbanisation | Semi-urban: areas within urban settlements but outside the municipal centre |  |
|  | Rural: areas outside major population centres and urban settlements |  |
| ^a^ Household income refers to an individual’s disposable income per consumption unit. Disposable income includes all taxable and tax-exempt income, minus taxes and negative transfers, and accounts for capital gains or losses—for example, from the sale of stocks, mutual funds, or real estate. To ensure comparability across different household types, Statistics Sweden applies a consumption-weighting system based on household composition. This system adjusts disposable income by dividing it by a household’s consumption weight, which reflects expected economic needs. The scale, informed by budget calculations from the Swedish Consumer Agency, assigns weights as follows: 1.0 for a single adult, 1.51 for a cohabiting couple, 0.6 for each additional adult, 0.52 for the first child (aged 0–19), and 0.42 for each subsequent child.  ^b^ Based on the distribution of individuals with a low household income living in Demographic Statistical Areas (DeSOs; n = 5984) in Sweden. Proportions of inhabitants with low household income were calculated for each DeSO, which were thereby sorted from least deprived DeSOs to most deprived. Based on this ranking we allocated each DeSO into national quintiles (Q1-Q5), each quintile comprising approximately 1197 different DeSOs. | | |

| Table S2. Clinical variables: liver diseases and comorbidity | |
| --- | --- |
| Aetiologies |  |
| Hepatitis B | ICD-10: B16, B17.0, B18.0, B18.1 *OR* specified as the main cause of HCC in SweLiv |
| Hepatitis C | ICD-10: B17.1, B18.2 *OR* specified as the main cause of HCC in SweLiv |
| ALD | ICD-10: K70 *OR* alcohol use disorder *AND* no other known liver disease, ICD-10: E24.4, F10, G31.2, G62.1, G72.1, I42.6, K29.2, K85.2, K86.0, R78.0, T51.0, T51.9, Y57.3, Y90, X65, Z71.4, Z72.1 |
|  | *OR* ATC: N07BB01 (disulfiram), N07BB03 (acamprosat), N07BB04 (naltrexon) *OR* specified as the main cause of HCC in SweLiv |
| MASLD | ICD-10: K75.8, K76.0 *OR* obesity (ICD-10: E66) *AND/OR* diabetes (as defined below) *OR* specified as the main cause of HCC in SweLiv |
| Rare liver diseases | ICD-10: K75.4, K83.0A, K74.3, E83.0B, E88.0A, E88.0B, E83.1, K73.9, K73.2, I82.0, K76.5, K74.4, K74.5 *OR* specified as the main cause of HCC in SweLiv |
| Cryptogenic cirrhosis | ICD-10: K74.6 *AND* no diagnosed liver disease *OR* specified as the main cause of HCC in SweLiv |
| Liver cirrhosis | ICD-10: B18.0E, B18.0G, B18.1E, B18.1G, B18.2E, B18.2G, B18.8E, B18.8G, B18.9E, B18.9G, G93.4, I85.0 I85.9, I98.2, I98.3, K70.3, K71.7, K74.6, K76.6, K76.7, R18. (no heart failure [I50, I11.0, I13.0, I13.2] or cancer other than HCC). Procedural: JCA20, JCA22, JCA32, JDA22 *OR* TJA10, TJA40 *AND* known liver disease *OR* specified as having cirrhosis in SweLiv |
| Child-Pugh score | Bilirubin (μmol/L): <34.2 (+1), 34.2-51.3 (+2), >51.3 (+3) |
|  | Albumin (g/L): >35 (+1), 28-35 (+2), <28 (+3) |
|  | Prothrombin time (international normalized ratio): <1.7 (+1), 1.7-2.2 (+2), >2.2 (+3); not used for patients treated with warfarin |
|  | Ascites: absent (+1), slight (+2), moderate (+3) |
|  | Encephalopathy: none (+1), grade 1-2 (+2), grade 3-4 (+3) |
|  | Calculation for missing data: |
|  | 1 laboratory parameter missing: CP ≤ 7 if the sum of other parameters = 4 |
|  | 2-3 laboratory parameters missing: CP > 7 if the sum of other parameters ≥ 6 |
|  | Encephalopathy grade missing: +1 if the sum of other parameters = 4 and ECOG = 0 |
|  | Ascites grade missing: +1 if the sum of other parameters =4 and ECOG =0 |
| Comorbidities |  |
| Diabetes | ICD-10: E10-E14; ATC: A10A (insulin), A10B (other glucose lowering drugs), A10X (other drugs against diabetes) |
| CAD | ICD-10: I20-I25; ATC: C01DA (organic nitrates) |
| CKD | ICD-10: N11, N18, I13.1, I13.2, I13.9, I12.0, Z94.0, T86.1, Z49, Z99.2 |
| COPD | ICD-10: J41-J44 (40 years and older) |
| Non-HCC cancer | All non-cancer ICD-10 codes registered within 5 years prior to HCC diagnosis |
| Alcohol use disorder | ICD-10: F10, E244, E52, G312, G621, G721, I426, K292, K852, K860, R780, T510, T519, Y573, Y90, Y91, X65, Z714, Z721 |
| Drug use disorder | ICD-10: F11-F16, F18, F19 |
| Depression or anxiety | ICD-10: F30-34, F38, F39, F41, F43 |
| ALD: alcohol-related liver disease; ATC: Anatomical Therapeutic Chemical Classification System; CAD: coronary artery disease; CKD: chronic kidney disease; COPD: chronic obstructive pulmonary disease; CP: Child-Pugh; ECOG PS: Eastern Cooperative Oncology Group performance status; HCC: hepatocellular carcinoma; ICD: International Classification of Diseases; MASLD: metabolic dysfunction-associated steatotic liver disease; SweLiv: Swedish quality register for cancers found in the liver, gallbladder and bile ducts. | |

| Table S3. Clinical variables: tumour related, performance status, and treatment | |
| --- | --- |
| Diagnostic pathway | Surveillance: patient with known liver disease included in the surveillance program for HCC |
|  | Clinical symptoms: diagnosed with HCC during a clinical work-up related to liver disease-associated symptoms |
|  | Incidental: HCC diagnosis on passant in surgery or radiology in a patient without clinical symptoms related to liver disease |
| Tumour size | Largest diameter in millimetres: ≤ 20, 21-30, >30 |
| Tumour number | Single, 2-3, >3 (multinodular) |
| Lymph node metastasis | TNM classification for HCC 8^th^ Edition: N0, N1, NX |
| Extrahepatic metastasis | TNM classification for HCC 8^th^ Edition: M0, M1, MX |
| Tumour thrombosis | Yes/No |
| Portal vein thrombosis | Yes/No |
| ECOG performance status | 0: Fully active, able to carry on all pre-disease performance without restriction. |
|  | 1: Restricted in physically strenuous activity but ambulatory and able to perform work of a light or sedentary nature. |
|  | 2: Ambulatory and capable of all self-care but unable to carry out any work activities; up and about >50% of waking hours. |
|  | 3: Capable of only limited self-care; confined to bed or chair >50% of waking hours. |
|  | 4: Completely disabled; cannot conduct any self-care; totally confined to bed or chair. |
| HCC Stage | Early: |
|  | 1) Single tumour or up to 3 nodules ≤3 cm *AND* |
|  | 2) N0, M0 *AND* |
|  | 3) ECOG PS 0-1 (2 in transplant/resection candidates) *AND* |
|  | 4) Child-Pugh score ≤7 (not necessary if transplant candidate) |
|  | Intermediate: |
|  | 1) Multinodular tumours *AND* |
|  | 2) N0, M0 *AND* |
|  | 3) ECOG PS 0-1 (2 in transplant/resection candidates) *AND* |
|  | 4) Child-Pugh score ≤7 (not necessary if transplant candidate) |
|  | Advanced: |
|  | 1) M1 *OR* N1 *AND* |
|  | 2) ECOG PS 0-1 (2 in transplant/resection candidates) *AND* |
|  | 3) Child-Pugh score ≤7 (not necessary if transplant candidate) |
|  | Terminal: |
|  | 1) Child-Pugh score >7 *OR* |
|  | 2) ECOG ≥ 2 *AND* |
|  | 3) No candidate for liver transplantation *AND* |
|  | 4) Severe comorbidity burden |
| Curative-intent treatment | Transplantation: if registered at any point, regardless of previous treatments. ICD-10 Z94.4, T86.4; Procedural: JCA20, JCA22, JCA32, JDA22, JJC |
|  | Resection: if performed before ablation. Procedural: JJB, JJA40, JJA41 |
|  | Ablation: if performed before resection. Procedural: JJA43, JJA44, TJJ10, |
| ATC: Anatomical Therapeutic Chemical Classification System; ECOG PS: Eastern Cooperative Oncology Group performance status; HCC: hepatocellular carcinoma; ICD: International Classification of Diseases; M: extrahepatic metastasis; N: regional lymph node metastasis; SweLiv: Swedish quality register for cancers found in the liver, gallbladder and bile ducts. | |

| Table S4. Sociodemographic and clinical characteristics of 1514 individuals with surveillance-detected hepatocellular carcinoma in Sweden, 2012–2022, stratified by sex | | | |
| --- | --- | --- | --- |
|  | | **Sex** | |
|  | | **Male** | **Female** |
| Individuals included, n (%) | | 1152 (76) | 362 (24) |
| Median age (IQR) |  | 66 (60–72) | 69 (62–75) |
| Age group | 18–59 | 279 (24) | 67 (19) |
|  | 60–69 | 473 (41) | 128 (35) |
|  | 70–79 | 348 (30) | 142 (39) |
|  | 80+ | 52 (5) | 25 (7) |
| Region of birth | Nordic | 949 (82) | 291 (81) |
|  | Europe (non-Nordic) | 79 (7) | 30 (8) |
|  | Non-European | 121 (11) | 41 (11) |
| Marital status | Married | 522 (45) | 135 (37) |
|  | Single | 300 (26) | 57 (16) |
|  | Divorced | 285 (25) | 118 (33) |
|  | Widowed | 45 (4) | 52 (14) |
| Educational | >12 years | 219 (19) | 77 (21) |
| attainment | 10–12 years | 549 (48) | 152 (42) |
|  | ≤9 years | 364 (31) | 120 (33) |
|  | Unknown | 20 (2) | 13 (4) |
| Household | High | 185 (16) | 51 (14) |
| income | Medium | 482 (42) | 143 (40) |
|  | Low | 485 (42) | 168 (46) |
| Neighbourhood | Q1 (least) | 150 (13) | 39 (11) |
| deprivation | Q2 | 178 (15) | 65 (18) |
|  | Q3 | 231 (20) | 75 (21) |
|  | Q4 | 238 (21) | 75 (21) |
|  | Q5 (most) | 355 (31) | 108 (30) |
| Degree of urbanisation | Urban | 879 (76) | 291 (80) |
|  | Semi-urban | 90 (8) | 25 (9) |
|  | Rural | 183 (16) | 46 (13) |
| Period | 2012–2015 | 354 (31) | 105 (29) |
|  | 2016­–2019 | 453 (39) | 151 (42) |
|  | 2020–2022 | 345 (30) | 106 (29) |
| Aetiology | HCV | 486 (42) | 214 (40) |
|  | ALD | 307 (27) | 138 (25) |
|  | HBV | 137 (12) | 49 (9) |
|  | MASLD | 111 (10) | 60 (11) |
|  | Rare liver diseases | 59 (5) | 46 (9) |
|  | Cryptogenic | 52 (4) | 33 (6) |
| CP score | ≤7 | 1007 (87) | 306 (85) |
|  | >7 | 145 (13) | 56 (15) |
| ECOG PS | 0–1 | 930 (81) | 279 (77) |
|  | ≥2 | 222 (19) | 83 (23) |
| Tumour size | Median (IQR) | 27 (20–40) | 25 (18–38) |
| (largest) | ≤20 mm | 388 (34) | 134 (37) |
|  | 21–30 mm | 311 (27) | 99 (27) |
|  | >30 mm | 453 (39) | 129 (36) |
| N of tumours | 1 | 657 (57) | 230 (63) |
|  | 2–3 | 346 (30) | 105 (29) |
|  | >3 | 141 (12) | 25 (7) |
|  | Uncertain | 8 (1) | 2 (1) |
| Thrombosis | Tumour | 56 (5) | 18 (5) |
|  | Portal vein (benign) | 106 (9) | 25 (7) |
| Metastasis | Extrahepatic | 38 (3) | 8 (2) |
| Stage at | Early | 627 (55) | 218 (60) |
| diagnosis | Intermediate | 300 (26) | 91 (25) |
|  | Advanced | 82 (7) | 22 (6) |
|  | Terminal | 143 (12) | 31 (9) |
| Comorbidity | Type 2 diabetes | 487 (42) | 139 (38) |
|  | CAD | 148 (13) | 38 (11) |
|  | CKD | 94 (8) | 26 (7) |
|  | COPD | 149 (13) | 45 (12) |
|  | Extrahepatic cancer^a^ | 152 (13) | 53 (15) |
|  | Alcohol use disorder | 274 (24) | 45 (12) |
|  | Drug use disorder | 105 (9) | 17 (5) |
|  | Depression/anxiety | 153 (13) | 53 (15) |

**Table S4. … continuation**

| Curative-intent | Transplantation | 116 (10) | 26 (7) |
| --- | --- | --- | --- |
| treatment | Resection | 197 (17) | 63 (17) |
|  | Ablation | 425 (37) | 147 (41) |
| ALD: alcohol-related liver disease; CAD: coronary artery disease; CKD: chronic kidney disease; COPD: chronic obstructive pulmonary disease; CP: Child-Pugh; ECOG: Eastern Cooperative Oncology Group Performance Status; HBV: hepatitis B virus; HCV: hepatitis C virus; IQR: interquartile range; MASLD: metabolic dysfunction-associated steatotic liver disease.  Rare liver diseases included primary sclerosing cholangitis, primary biliary cholangitis, and autoimmune hepatitis.  ^a^ Extrahepatic cancers diagnosed within 5 years prior to hepatocellular carcinoma diagnosis. | | | |

| Table S5. Sociodemographic and clinical characteristics of 1514 individuals with surveillance-detected hepatocellular carcinoma in Sweden, 2012–2022, stratified by region of birth | | | | |
| --- | --- | --- | --- | --- |
|  | | **Region of birth** | | |
|  | | **Nordic** | **Europe (non-Nordic)** | **Non-European** |
| Individuals included | | 1240 (82) | 109 (7) | 165 (11) |
| Sex | Male | 949 (77) | 79 (73) | 124 (75) |
|  | Female | 291 (23) | 30 (27) | 41 (25) |
| Median age (IQR) |  | 67 (61–73) | 65 (58–72) | 61 (55–70) |
| Age group | 18–59 | 236 (19) | 35 (32) | 72 (44) |
|  | 60–69 | 513 (41) | 37 (34) | 51 (31) |
|  | 70–79 | 422 (34) | 33 (30) | 35 (21) |
|  | 80+ | 66 (5) | 4 (4) | 7 (4) |
| Marital status | Married | 502 (40) | 64 (59) | 91 (55) |
|  | Single | 332 (27) | 8 (7) | 17 (10) |
|  | Divorced | 328 (26) | 27 (25) | 48 (29) |
|  | Widowed | 78 (6) | 10 (9) | 9 (6) |
| Educational | >12 years | 226 (18) | 30 (27) | 40 (24) |
| attainment | 10–12 years | 616 (50) | 35 (32) | 50 (30) |
|  | ≤9 years | 391 (31) | 41 (38) | 52 (32) |
|  | Unknown | 7 (1) | 3 (3) | 23 (14) |
| Household | High | 211 (17) | 10 (9) | 15 (9) |
| income | Medium | 535 (43) | 34 (31) | 56 (34) |
|  | Low | 494 (40) | 65 (60) | 94 (57) |
| Neighbourhood | Q1 (least) | 171 (14) | 9 (8) | 9 (5) |
| deprivation | Q2 | 216 (17) | 13 (12) | 14 (9) |
|  | Q3 | 263 (21) | 18 (16) | 25 (15) |
|  | Q4 | 269 (22) | 16 (15) | 28 (17) |
|  | Q5 (most) | 321 (26) | 53 (49) | 89 (54) |
| Degree of | Urban | 916 (74) | 97 (89) | 157 (95) |
| urbanisation | Semi-urban | 106 (9) | 4 (4) | 5 (3) |
|  | Rural | 218 (17) | 8 (7) | 3 (2) |
| Period | 2012–2015 | 372 (30) | 39 (36) | 48 (29) |
|  | 2016­–2019 | 490 (39) | 41 (38) | 73 (44) |
|  | 2020–2022 | 378 (31) | 29 (26) | 44 (27) |
| Aetiology | HCV | 513 (41) | 42 (39) | 54 (33) |
|  | ALD | 327 (26) | 19 (17) | 18 (11) |
|  | HBV | 81 (7) | 31 (28) | 71 (43) |
|  | MASLD | 136 (11) | 10 (9) | 14 (8) |
|  | Rare liver diseases | 123 (10) | 3 (3) | 6 (4) |
|  | Cryptogenic | 60 (5) | 4 (4) | 2 (1) |
| CP score | ≤7 | 1083 (87) | 93 (85) | 137 (83) |
|  | >7 | 157 (13) | 16 (15) | 28 (17) |
| ECOG PS | 0–1 | 979 (79) | 88 (81) | 142 (86) |
|  | ≥2 | 261 (21) | 21 (19) | 23 (14) |
| Tumour size | Median (IQR) | 26 (20–40) | 24 (16–35) | 25 (20–40) |
| (largest) | ≤20 mm | 418 (34) | 51 (47) | 53 (32) |
|  | 21–30 mm | 338 (27) | 23 (21) | 49 (30) |
|  | >30 mm | 484 (39) | 35 (32) | 63 (38) |
| N of tumours | 1 | 720 (58) | 60 (55) | 107 (65) |
|  | 2–3 | 369 (30) | 36 (33) | 46 (28) |
|  | >3 | 142 (11) | 13 (12) | 11 (6) |
|  | Uncertain | 9 (1) | 0 | 1 (1) |
| Thrombosis | Tumour | 60 (5) | 4 (4) | 10 (6) |
|  | Portal vein (benign) | 107 (9) | 9 (8) | 15 (9) |
| Metastasis | Extrahepatic | 39 (3) | 2 (2) | 5 (3) |
| Stage at | Early | 686 (55) | 57 (52) | 102 (62) |
| diagnosis | Intermediate | 318 (26) | 36 (33) | 37 (22) |
|  | Advanced | 81 (7) | 9 (8) | 14 (9) |
|  | Terminal | 155 (12) | 7 (7) | 12 (7) |
| Comorbidity | Type 2 diabetes | 514 (41) | 45 (41) | 67 (41) |
|  | CAD | 161 (13) | 11 (10) | 14 (9) |
|  | CKD | 99 (8) | 8 (7) | 13 (8) |
|  | COPD | 172 (14) | 12 (11) | 10 (6) |
|  | Extrahepatic cancer^a^ | 185 (15) | 11 (10) | 9 (5) |
|  | Alcohol use disorder | 287 (23) | 21 (19) | 11 (7) |
|  | Drug use disorder | 111 (9) | 4 (4) | 7 (4) |
|  | Depression/anxiety | 172 (14) | 14 (13) | 20 (12) |

**Table S5. … continuation**

| Curative-intent | Transplantation | 100 (8) | 14 (13) | 28 (17) |
| --- | --- | --- | --- | --- |
| treatment | Resection | 209 (17) | 15 (14) | 36 (22) |
|  | Ablation | 474 (38) | 47 (43) | 51 (31) |
| ALD: alcohol-related liver disease; CAD: coronary artery disease; CKD: chronic kidney disease; COPD: chronic obstructive pulmonary disease; CP: Child-Pugh; ECOG: Eastern Cooperative Oncology Group Performance Status; HBV: hepatitis B virus; HCV: hepatitis C virus; IQR: interquartile range; MASLD: metabolic dysfunction-associated steatotic liver disease.  Rare liver diseases included primary sclerosing cholangitis, primary biliary cholangitis, and autoimmune hepatitis.  ^a^ Extrahepatic cancers diagnosed within 5 years prior to hepatocellular carcinoma diagnosis. | | | | |

| Table S6. Sociodemographic and clinical characteristics of 1514 individuals with surveillance-detected hepatocellular carcinoma in Sweden, 2012–2022, stratified by household income | | | | |
| --- | --- | --- | --- | --- |
|  | | **Household income** | | |
|  | | **Low** | **Medium** | **High** |
| Individuals included | | 625 (41) | 653 (43) | 236 (16) |
| Sex | Male | 485 (74) | 482 (77) | 185 (78) |
|  | Female | 168 (26) | 143 (23) | 51 (22) |
| Median age (IQR) |  | 65 (59–72) | 67 (61–73) | 67 (61–72) |
| Age group | 18–59 | 184 (28) | 120 (19) | 42 (18) |
|  | 60–69 | 235 (36) | 264 (42) | 102 (43) |
|  | 70–79 | 210 (32) | 196 (32) | 84 (36) |
|  | 80+ | 24 (4) | 45 (7) | 8 (3) |
| Region of birth | Nordic | 494 (76) | 535 (86) | 211 (90) |
|  | Europe (non-Nordic) | 65 (10) | 34 (5) | 10 (4) |
|  | Non-European | 94 (14) | 56 (9) | 15 (6) |
| Marital status | Married | 169 (26) | 332 (53) | 156 (66) |
|  | Single | 208 (32) | 116 (19) | 33 (14) |
|  | Divorced | 230 (35) | 138 (22) | 35 (15) |
|  | Widowed | 46 (7) | 39 (6) | 12 (5) |
| Educational | >12 years | 70 (11) | 132 (21) | 94 (40) |
| attainment | 10–12 years | 308 (47) | 299 (48) | 94 (40) |
|  | ≤9 years | 250 (38) | 188 (30) | 46 (19) |
|  | Unknown | 25 (4) | 6 (1) | 2 (1) |
| Neighbourhood | Q1 (least) | 34 (5) | 88 (14) | 67 (29) |
| deprivation | Q2 | 86 (13) | 109 (17) | 48 (20) |
|  | Q3 | 104 (16) | 146 (23) | 56 (24) |
|  | Q4 | 143 (22) | 141 (23) | 29 (12) |
|  | Q5 (most) | 286 (44) | 141 (23) | 36 (15) |
| Degree of | Urban | 503 (77) | 472 (76) | 195 (83) |
| urbanisation | Semi-urban | 51 (8) | 52 (8) | 12 (5) |
|  | Rural | 99 (15) | 101 (16) | 29 (12) |
| Period | 2012–2015 | 185 (28) | 194 (31) | 80 (34) |
|  | 2016­–2019 | 253 (39) | 259 (41) | 92 (39) |
|  | 2020–2022 | 215 (33) | 172 (28) | 64 (27) |
| Aetiology | HCV | 324 (49) | 214 (34) | 71 (30) |
|  | ALD | 109 (17) | 180 (29) | 75 (32) |
|  | HBV | 108 (17) | 60 (10) | 15 (6.5) |
|  | MASLD | 52 (8) | 77 (12) | 31 (13) |
|  | Rare liver diseases | 40 (6) | 63 (10) | 29 (12) |
|  | Cryptogenic | 20 (3) | 31 (5) | 15 (6.5) |
| CP score | ≤7 | 560 (86) | 548 (88) | 205 (87) |
|  | >7 | 93 (14) | 77 (12) | 31 (13) |
| ECOG PS | 0–1 | 490 (75) | 521 (83) | 198 (84) |
|  | ≥2 | 163 (25) | 104 (17) | 38 (16) |
| Tumour size | Median (IQR) | 28 (20–40) | 26 (18–40) | 25 (20–39) |
| (largest) | ≤20 mm | 208 (32) | 231 (37) | 83 (35) |
|  | 21–30 mm | 182 (28) | 158 (25) | 70 (30) |
|  | >30 mm | 263 (40) | 236 (38) | 83 (35) |
| N of tumours | 1 | 375 (58) | 375 (60) | 137 (58) |
|  | 2–3 | 199 (30) | 188 (30) | 64 (27.1) |
|  | >3 | 73 (11) | 60 (10) | 33 (14) |
|  | Uncertain | 6 (1) | 2 (<1) | 2 (0.8) |
| Thrombosis | Tumour | 39 (6) | 28 (4) | 7 (3) |
|  | Portal vein (benign) | 66 (10) | 52 (8) | 13 (6) |
| Metastasis | Extrahepatic | 24 (4) | 16 (3) | 6 (3) |
| Stage at | Early | 335 (51) | 370 (59) | 140 (59) |
| diagnosis | Intermediate | 166 (25) | 163 (26) | 62 (26) |
|  | Advanced | 50 (8) | 39 (6) | 15 (7) |
|  | Terminal | 102 (16) | 53 (9) | 19 (8) |
| Comorbidity | Type 2 diabetes | 252 (39) | 274 (44) | 100 (42) |
|  | CAD | 81 (12) | 81 (13) | 24 (10) |
|  | CKD | 52 (8) | 48 (8) | 20 (8) |
|  | COPD | 105 (16) | 70 (11) | 19 (8) |
|  | Extrahepatic cancer^a^ | 71 (11) | 97 (16) | 37 (16) |
|  | Alcohol use disorder | 171 (26) | 115 (18) | 33 (14) |
|  | Drug use disorder | 80 (12) | 34 (5) | 8 (3) |
|  | Depression/anxiety | 107 (16) | 66 (11) | 33 (14) |

**Table S6. … continuation**

| Curative-intent | Transplantation | 50 (8) | 59 (9) | 33 (14) |
| --- | --- | --- | --- | --- |
| treatment | Resection | 104 (16) | 111 (18) | 45 (19) |
|  | Ablation | 232 (35) | 242 (39) | 98 (42) |
| ALD: alcohol-related liver disease; CAD: coronary artery disease; CKD: chronic kidney disease; COPD: chronic obstructive pulmonary disease; CP: Child-Pugh; ECOG: Eastern Cooperative Oncology Group Performance Status; HBV: hepatitis B virus; HCV: hepatitis C virus; IQR: interquartile range; MASLD: metabolic dysfunction-associated steatotic liver disease.  Rare liver diseases included primary sclerosing cholangitis, primary biliary cholangitis, and autoimmune hepatitis.  ^a^ Extrahepatic cancers diagnosed within 5 years prior to hepatocellular carcinoma diagnosis. | | | | |

| Table S7. Factors associated with the likelihood of curative-intent treatment receipt among 1514 individuals with surveillance-detected hepatocellular carcinoma in Sweden, 2012–2022 (individual-level socioeconomic status) | | | |
| --- | --- | --- | --- |
|  | | **Multivariable** | |
|  | | **aOR (95% CI)** | **P-value** |
| Sex | Male | 1.0 (ref) | - |
|  | Female | 0.97 (0.69–1.35) | 0.839 |
| Age group | 18–59 | 1.0 (ref) | - |
|  | 60–69 | 0.98 (0.67–1.44) | 0.916 |
|  | 70–79 | 0.60 (0.39–0.93) | 0.023 |
|  | 80+ | 0.35 (0.17–0.71) | 0.003 |
| Region of birth | Nordic | 1.0 (ref) | - |
|  | Europe (non-Nordic) | 1.12 (0.63–1.98) | 0.705 |
|  | Non-European | 0.97 (0.57–1.63) | 0.895 |
| Marital status | Married | 1.0 (ref) | - |
|  | Single | 0.92 (0.63–1.36) | 0.676 |
|  | Divorced | 0.97 (0.68–1.39) | 0.867 |
|  | Widowed | 0.81 (0.46–1.42) | 0.455 |
| Educational | >12 years | 1.0 (ref) | - |
| attainment | 10–12 years | 1.03 (0.71–1.49) | 0.894 |
|  | ≤9 years | 1.17 (0.78–1.75) | 0.438 |
| Household income | High | 1.0 (ref) | - |
|  | Medium | 0.53 (0.34–0.81) | 0.003 |
|  | Low | 0.42 (0.26–0.66) | <0.001 |
| Period | 2012–2015 | 1.0 (ref) | - |
|  | 2016­–2019 | 0.91 (0.66–1.27) | 0.580 |
|  | 2020–2022 | 0.87 (0.60–1.26) | 0.471 |
| Aetiology | HCV | 1.0 (ref) | - |
|  | ALD | 0.98 (0.67–1.43) | 0.903 |
|  | HBV | 2.26 (1.35–3.77) | 0.002 |
|  | MASLD | 1.16 (0.69–1.95) | 0.576 |
|  | Rare liver diseases | 1.39 (0.81–2.38) | 0.233 |
|  | Cryptogenic | 1.01 (0.51–1.98) | 0.979 |
| CP score | ≤7 | 1.0 (ref) | - |
|  | >7 | 0.37 (0.25–0.56) | <0.001 |
| ECOG PS | 0–1 | 1.0 (ref) | - |
|  | ≥2 | 0.25 (0.17–0.35) | <0.001 |
| Tumour size | ≤20 mm | 1.0 (ref) | - |
| (largest) | 21–30 mm | 0.55 (0.38–0.79) | 0.001 |
|  | >30 mm | 0.14 (0.10–0.19) | <0.001 |
| N of tumours | 1 | 1.0 (ref) | - |
|  | 2–3 | 0.62 (0.46–0.83) | 0.002 |
|  | >3 | 0.14 (0.09–0.22) | <0.001 |
| Benign PVT |  | 0.20 (0.12–0.33) | <0.001 |
| Comorbidity | Type 2 diabetes | 1.05 (0.77–1.42) | 0.759 |
|  | CAD | 0.89 (0.60–1.31) | 0.543 |
|  | CKD | 1.34 (0.81–2.23) | 0.249 |
|  | COPD | 0.79 (0.53–1.16) | 0.230 |
|  | Extrahepatic cancer^a^ | 1.20 (0.80–1.78) | 0.380 |
|  | Alcohol use disorder | 1.09 (0.75–1.56) | 0.660 |
|  | Drug use disorder | 0.73 (0.43–1.24) | 0.242 |
|  | Depression/anxiety | 1.00 (0.66–1.52) | 0.999 |
| The multivariable logistic regression model, which included all the variables in this table, was statistically significant compared to the null model (Chi-square (36) = 575.70, p<0.001) and correctly classified 83% of cases.  ALD: alcohol-related liver disease; CAD: coronary artery disease; CI: confidence interval; CKD: chronic kidney disease; COPD: chronic obstructive pulmonary disease; CP: Child-Pugh; ECOG: Eastern Cooperative Oncology Group Performance Status; HBV: hepatitis B virus; HCV: hepatitis C virus; MASLD: metabolic dysfunction-associated steatotic liver disease; OR: odds ratio. PVT: benign portal vein thrombosis. Rare liver diseases included primary sclerosing cholangitis, primary biliary cholangitis, and autoimmune hepatitis.  ^a^ Extrahepatic cancers diagnosed within 5 years prior to hepatocellular carcinoma diagnosis. | | | |

| Table S8. Factors associated with the likelihood of curative-intent treatment receipt among 1514 individuals with surveillance-detected hepatocellular carcinoma in Sweden, 2012–2022 (contextual-level socioeconomic status) | | | |
| --- | --- | --- | --- |
|  | | **Multivariable** | |
|  | | **aOR (95% CI)** | **P-value** |
| Sex | Male | 1.0 (ref) | - |
|  | Female | 0.93 (0.68–1.28) | 0.671 |
| Age group | 18–59 | 1.0 (ref) | - |
|  | 60–69 | 0.97 (0.67–1.40) | 0.861 |
|  | 70–79 | 0.55 (0.36–0.85) | 0.006 |
|  | 80+ | 0.31 (0.16–0.60) | 0.001 |
| Neighbourhood | Q1 (least) | 1.0 (ref) | - |
| deprivation | Q2 | 0.60 (0.36–1.01) | 0.053 |
|  | Q3 | 0.66 (0.40–1.08) | 0.099 |
|  | Q4 | 0.51 (0.31–0.84) | 0.008 |
|  | Q5 | 0.56 (0.35–0.91) | 0.018 |
| Degree of urbanisation | Urban | 1.0 (ref) | - |
|  | Semi-urban | 1.09 (0.66–1.80) | 0.735 |
|  | Rural | 0.85 (0.58–1.24) | 0.404 |
| Period | 2012–2015 | 1.0 (ref) | - |
|  | 2016­–2019 | 0.88 (0.64–1.22) | 0.459 |
|  | 2020–2022 | 0.88 (0.61–1.26) | 0.477 |
| Aetiology | HCV | 1.0 (ref) | - |
|  | ALD | 0.88 (0.60–1.30) | 0.516 |
|  | HBV | 1.93 (1.21–3.10) | 0.006 |
|  | MASLD | 1.09 (0.65–1.81) | 0.755 |
|  | Rare liver diseases | 1.26 (0.74–2.15) | 0.396 |
|  | Cryptogenic | 1.04 (0.54–2.01) | 0.907 |
| CP score | ≤7 | 1.0 (ref) | - |
|  | >7 | 0.37 (0.25–0.56) | <0.001 |
| ECOG PS | 0–1 | 1.0 (ref) | - |
|  | ≥2 | 0.25 (0.18–0.35) | <0.001 |
| Tumour size | ≤20 mm | 1.0 (ref) | - |
| (largest) | 21–30 mm | 0.53 (0.36–0.76) | 0.001 |
|  | >30 mm | 0.14 (0.10–0.19) | <0.001 |
| N of tumours | 1 | 1.0 (ref) | - |
|  | 2–3 | 0.64 (0.48–0.86) | 0.003 |
|  | >3 | 0.14 (0.09–0.23) | <0.001 |
| Benign PVT |  | 0.18 (0.11–0.30) | <0.001 |
| Comorbidity | Type 2 diabetes | 1.09 (0.81–1.46) | 0.573 |
|  | CAD | 0.87 (0.59–1.28) | 0.484 |
|  | CKD | 1.35 (0.82–2.22) | 0.236 |
|  | COPD | 0.78 (0.53–1.14) | 0.200 |
|  | Extrahepatic cancer^a^ | 1.23 (0.83–1.82) | 0.303 |
|  | Alcohol use disorder | 1.01 (0.71–1.45) | 0.941 |
|  | Drug use disorder | 0.67 (0.40–1.14) | 0.140 |
|  | Depression/anxiety | 0.96 (0.64–1.45) | 0.855 |
| The multivariable logistic regression model, which included all the variables in this table, was statistically significant compared to the null model (Chi-square (35) = 571.74, p<0.001) and correctly classified 79% of cases.  ALD: alcohol-related liver disease; CAD: coronary artery disease; CI: confidence interval; CKD: chronic kidney disease; COPD: chronic obstructive pulmonary disease; CP: Child-Pugh; ECOG: Eastern Cooperative Oncology Group Performance Status; HBV: hepatitis B virus; HCV: hepatitis C virus; MASLD: metabolic dysfunction-associated steatotic liver disease; OR: odds ratio. PVT: benign portal vein thrombosis. Rare liver diseases included primary sclerosing cholangitis, primary biliary cholangitis, and autoimmune hepatitis.  ^a^ Extrahepatic cancers diagnosed within 5 years prior to hepatocellular carcinoma diagnosis. | | | |

| Table S9. Post curative-intent treatment survival probabilities and median survival among 974 individuals with surveillance-detected hepatocellular carcinoma in Sweden, 2012–2022 | | | | | |
| --- | --- | --- | --- | --- | --- |
|  |  |  | **Survival probability**  **(95% CI)** | |  |
|  | **N** | **Deaths (%)^a^** | **1-year** | **5-year** | **Median survival in years (95% CI)** |
| Overall | 974 | 438 (45) | 0.92 (0.90–0.94) | 0.56 (0.52–0.59) | 5.98 (5.39–6.84) |
| Sex |  |  |  |  |  |
| Male | 531 | 326 (44) | 0.93 (0.90–0.94) | 0.57 (0.53–0.61) | 6.30 (5.67–7.51) |
| Female | 438 | 112 (47) | 0.91 (0.87–0.94) | 0.51 (0.43–0.58) | 5.26 (4.03–6.64) |
| Age group |  |  |  |  |  |
| 18–59 | 239 | 88 (37) | 0.93 (0.89–0.95) | 0.69 (0.62–0.75) | Not reached |
| 60–69 | 410 | 165 (40) | 0.93 (0.90–0.95) | 0.61 (0.56–0.66) | 7.49 (5.95–10.2) |
| 70–79 | 285 | 160 (56) | 0.91 (0.88–0.94) | 0.38 (0.31–0.45) | 3.92 (3.33–4.52) |
| 80+ | 40 | 25 (63) | 0.87 (0.73–0.95) | 0.17 (0.05–0.35) | 2.65(1.97–3.55) |
| Region of birth |  |  |  |  |  |
| Nordic | 783 | 371 (47) | 0.92 (0.90–0.94) | 0.54 (0.50–0.58) | 5.80 (4.96–6.31) |
| Europe (non-Nordic) | 76 | 23 (30) | 0.95 (0.86–0.98) | 0.66 (0.53–0.77) | Not reached |
| Non-European | 115 | 44 (38) | 0.90 (0.83–0.94) | 0.61 (0.50–0.70) | 8.18 (5.64–) |
| Marital status |  |  |  |  |  |
| Married | 434 | 196 (45) | 0.93 (0.90–0.95) | 0.54 (0.48–0.59) | 6.50 (4.83–7.91) |
| Single | 228 | 96 (42) | 0.91 (0.86–0.94) | 0.65 (0.57–0.71) | 6.04 (5.80–10.2) |
| Divorced | 256 | 112 (44) | 0.94 (0.91–0.97) | 0.53 (0.46–0.60) | 5.64 (4.68–8.31) |
| Widowed | 56 | 34 (61) | 0.87 (0.75–0.94) | 0.43 (0.29–0.56) | 3.81 (3.18–6.31) |
| Educational attainment |  |  |  |  |  |
| >12 years | 201 | 164 (42) | 0.91 (0.87–0.95) | 0.57 (0.49–0.65) | 5.89 (4.97–7.91) |
| 10–12 years | 446 | 430 (46) | 0.93 (0.90–0.95) | 0.56 (0.51–0.61) | 6.31 (5.36–7.87) |
| ≤9 years | 305 | 309 (47) | 0.92 (0.88–0.95) | 0.54 (0.47–0.60) | 5.79 (4.65–6.96) |
| Household income |  |  |  |  |  |
| High | 176 | 76 (43) | 0.94 (0.89–0.97) | 0.59 (0.50–0.66) | 7.03 (5.01-9.05) |
| Medium | 412 | 178 (43) | 0.92 (0.89–0.94) | 0.60 (0.54–0.65) | 6.84 (5.89–8.59) |
| Low | 386 | 184 (48) | 0.92 (0.89–0.95) | 0.49 (0.43–0.55) | 4.95 (4.29–5.86) |
| Neighbourhood deprivation |  |  |  |  |  |
| Q1 (least) | 135 | 72 (53) | 0.93 (0.87–0.96) | 0.52 (0.43–0.61) | 5.09 (3.97–6.50) |
| Q2 | 151 | 60 (40) | 0.92 (0.86–0.95) | 0.58 (0.49–0.67) | 7.48 (4.78–) |
| Q3 | 206 | 90 (44) | 0.92 (0.88–0.95) | 0.59 (0.51–0.66) | 6.96 (5.20–) |
| Q4 | 192 | 92 (48) | 0.92 (0.87–0.95) | 0.50 (0.42–0.58) | 4.81 (3.81–6.84) |
| Q5 (most) | 290 | 124 (43) | 0.93 (0.89–0.95) | 0.57 (0.50–0.63) | 6.04 (5.15–8.85) |
| Degree of urbanisation |  |  |  |  |  |
| Urban | 759 | 332 (44) | 0.93 (0.91–0.94) | 0.57 (0.53–0.61) | 6.50 (5.64–7.56) |
| Semi-urban | 77 | 40 (52) | 0.92 (0.83–0.96) | 0.49 (0.35–0.61) | 4.68 (3.84–6.77) |
| Rural | 138 | 66 (48) | 0.90 (0.83–0.94) | 0.52 (0.42–0.60) | 5.42 (4.51–10.7) |
| CI: confidence interval. All individuals were followed until death, emigration, or 24 May 2024, whichever occurred first.  ^a^ Deaths at the end of follow-up. | | | | | |

| Table S10. Multivariable estimates for post curative-intent treatment mortality among 974 individuals with surveillance-detected hepatocellular carcinoma in Sweden, 2012–2022 (contextual-level socioeconomic status) | | | |
| --- | --- | --- | --- |
|  | | **Multivariable** | |
|  | | **aHR (95% CI)** | **P-value** |
| Sex | Male | 1.0 (ref) | - |
|  | Female | 1.10 (0.88–1.39) | 0.397 |
| Age group | 18–59 | 1.0 (ref) | - |
|  | 60–69 | 1.13 (0.85–1.49) | 0.383 |
|  | 70–79 | 1.64 (1.20–2.24) | 0.002 |
|  | 80+ | 2.63 (1.58–4.36) | <0.001 |
| Neighbourhood | Q1 (least) | 1.0 (ref) | - |
| deprivation | Q2 | 0.78 (0.55–1.11) | 0.178 |
|  | Q3 | 1.02 (0.73–1.41) | 0.926 |
|  | Q4 | 1.18 (0.85–1.65) | 0.314 |
|  | Q5 (most) | 1.13 (0.83–1.53) | 0.438 |
| Degree of urbanisation | Urban | 1.0 (ref) | 0.759 |
|  | Semi-urban | 1.40 (0.99–1.96) | 0.053 |
|  | Rural | 1.14 (0.86–1.51) | 0.374 |
| Period | 2012–2015 | 1.0 (ref) | - |
|  | 2016­–2019 | 0.77 (0.62–0.97) | 0.028 |
|  | 2020–2022 | 0.81 (0.60–1.09) | 0.166 |
| Aetiology | HCV | 1.0 (ref) | - |
|  | ALD | 1.54 (1.17–2.02) | 0.002 |
|  | HBV | 1.03 (0.74–1.43) | 0.858 |
|  | MASLD | 1.33 (0.93–1.90) | 0.116 |
|  | Rare liver diseases | 1.04 (0.69–1.57) | 0.836 |
|  | Cryptogenic | 1.54 (0.89–2.66) | 0.121 |
| Comorbidity | Type 2 diabetes | 1.04 (0.84–1.29) | 0.722 |
|  | CAD | 1.15 (0.87–1.53) | 0.334 |
|  | CKD | 1.73 (1.26–2.37) | 0.001 |
|  | COPD | 1.40 (1.06–1.86) | 0.019 |
|  | Extrahepatic cancer^a^ | 1.23 (0.95–1.61) | 0.120 |
|  | Alcohol use disorder | 1.22 (0.95–1.56) | 0.124 |
|  | Drug use disorder | 1.11 (0.76–1.64) | 0.580 |
|  | Depression/anxiety | 0.92 (0.69–1.23) | 0.103 |
| Treatment | Transplantation | 1.0 (ref) | - |
|  | Resection | 3.01 (1.98–4.58) | <0.001 |
|  | Ablation | 3.80 (2.56–5.64) | <0.001 |
| Results from a multivariable Cox regression model including all the variables in this table. Each individual was followed-up from the date of the first curative-intent treatment (transplantation, resection, or ablation) until date of death, emigration, or 24 May 2024, whichever occurred first.  ALD: alcohol-related liver disease; CAD: coronary artery disease; CI: confidence interval; CKD: chronic kidney disease; COPD: chronic obstructive pulmonary disease; HBV: hepatitis B virus; HCV: hepatitis C virus; HR: hazard ratio; MASLD: metabolic dysfunction-associated steatotic liver disease; Rare liver diseases included primary sclerosing cholangitis, primary biliary cholangitis, and autoimmune hepatitis.  ^a^ Extrahepatic cancers diagnosed within 5 years prior to hepatocellular carcinoma diagnosis. | | | |

| Table S11. Multivariable estimates for post-ablation mortality among 572 individuals with surveillance-detected hepatocellular carcinoma in Sweden, 2012–2022 | | | |
| --- | --- | --- | --- |
|  | | **Multivariable** | |
|  | | **aHR (95% CI)** | **P-value** |
| Sex | Male | 1.0 (ref) | - |
|  | Female | 1.02 (0.75–1.40) | 0.877 |
| Age group | 18–59 | 1.0 (ref) | - |
|  | 60–69 | 1.30 (0.90–1.87) | 0.164 |
|  | 70–79 | 2.00 (1.34–2.99) | 0.001 |
|  | 80+ | 3.27 (1.79–5.96) | <0.001 |
| Region of birth | Nordic | 1.0 (ref) | - |
|  | Europe (non-Nordic) | 0.69 (0.41–1.16) | 0.163 |
|  | Non-European | 0.99 (0.60–1.65) | 0.987 |
| Marital status | Married | 1.0 (ref) | - |
|  | Single | 1.05 (0.73–1.50) | 0.799 |
|  | Divorced | 1.02 (0.73–1.44) | 0.881 |
|  | Widowed | 1.17 (0.73–1.89) | 0.510 |
| Educational | >12 years | 1.0 (ref) | - |
| attainment | 10–12 years | 0.82 (0.59–1.15) | 0.257 |
|  | ≤9 years | 0.84 (0.59–1.21) | 0.345 |
| Household income | High | 1.0 (ref) | - |
|  | Medium | 0.97 (0.68–1.39) | 0.886 |
|  | Low | 1.51 (1.03–2.21) | 0.035 |
| Period | 2012–2015 | 1.0 (ref) | - |
|  | 2016­–2019 | 0.73 (0.55–0.98) | 0.035 |
|  | 2020–2022 | 0.70 (0.48–1.02) | 0.065 |
| Aetiology | HCV | 1.0 (ref) | - |
|  | ALD | 1.64 (1.17–2.29) | 0.004 |
|  | HBV | 1.21 (0.77–1.89) | 0.404 |
|  | MASLD | 1.44 (0.90–2.31) | 0.125 |
|  | Rare liver diseases | 1.10 (0.66–1.84) | 0.704 |
|  | Cryptogenic | 1.30 (0.38–4.39) | 0.677 |
| Comorbidity | Type 2 diabetes | 1.12 (0.86–1.46) | 0.405 |
|  | CAD | 1.05 (0.73–1.50) | 0.800 |
|  | CKD | 1.49 (0.96–2.31) | 0.077 |
|  | COPD | 1.51 (1.06–2.15) | 0.024 |
|  | Extrahepatic cancer^a^ | 1.22 (0.87–1.72) | 0.247 |
|  | Alcohol use disorder | 1.13 (0.89–1.47) | 0.325 |
|  | Drug use disorder | 0.73 (0.48–1.13) | 0.157 |
|  | Depression/anxiety | 0.77 (0.55–1.05) | 0.093 |
| Results from a multivariable Cox regression model including all the variables in this table. Each individual was followed up from the date of ablation until date of death, emigration, or 24 May 2024, whichever occurred first.  ALD: alcohol-related liver disease; CAD: coronary artery disease; CI: confidence interval; CKD: chronic kidney disease; COPD: chronic obstructive pulmonary disease; HBV: hepatitis B virus; HCV: hepatitis C virus; HR: hazard ratio; MASLD: metabolic dysfunction-associated steatotic liver disease; Rare liver diseases included primary sclerosing cholangitis, primary biliary cholangitis, and autoimmune hepatitis.  ^a^ Extrahepatic cancers diagnosed within 5 years prior to hepatocellular carcinoma diagnosis. | | | |

| Table S12. Multivariable estimates for post-resection mortality among 260 individuals with surveillance-detected hepatocellular carcinoma in Sweden, 2012–2022 | | | |
| --- | --- | --- | --- |
|  | | **Multivariable** | |
|  | | **aHR (95% CI)** | **P-value** |
| Sex | Male | 1.0 (ref) | - |
|  | Female | 0.92 (0.57–1.48) | 0.738 |
| Age group | 18–59 | 1.0 (ref) | - |
|  | 60–69 | 0.94 (0.51–1.72) | 0.831 |
|  | 70–79 | 1.22 (0.58–2.57) | 0.592 |
|  | 80+ | 2.04 (0.51–8.12) | 0.311 |
| Region of birth | Nordic | 1.0 (ref) | - |
|  | Europe (non-Nordic) | 0.48 (0.19–1.24) | 0.132 |
|  | Non-European | 1.02 (0.48–2.18) | 0.950 |
| Marital status | Married | 1.0 (ref) | - |
|  | Single | 0.93 (0.52–1.69) | 0.824 |
|  | Divorced | 1.12 (0.63–1.98) | 0.696 |
|  | Widowed | 0.99 (0.40–2.41) | 0.974 |
| Educational | >12 years | 1.0 (ref) | - |
| attainment | 10–12 years | 0.92 (0.51–1.65) | 0.784 |
|  | ≤9 years | 1.57 (0.87–2.83) | 0.137 |
| Household income | High | 1.0 (ref) | - |
|  | Medium | 1.23 (0.68–2.22) | 0.498 |
|  | Low | 1.29 (0.64–2.61) | 0.479 |
| Period | 2012–2015 | 1.0 (ref) | - |
|  | 2016­–2019 | 0.87 (0.55–1.37) | 0.552 |
|  | 2020–2022 | 1.06 (0.57–1.95) | 0.861 |
| Aetiology | HCV | 1.0 (ref) | - |
|  | ALD | 1.36 (0.71–2.59) | 0.349 |
|  | HBV | 0.87 (0.43–1.76) | 0.699 |
|  | MASLD | 1.14 (0.59–2.21) | 0.697 |
|  | Rare liver diseases | 1.16 (0.49–2.73) | 0.726 |
|  | Cryptogenic | 1.80 (0.82–3.97) | 0.143 |
| Comorbidity | Type 2 diabetes | 1.07 (0.66–1.74) | 0.790 |
|  | CAD | 1.80 (1.02–3.16) | 0.041 |
|  | CKD | 1.87 (0.98–3.57) | 0.059 |
|  | COPD | 1.74 (0.95–3.19) | 0.071 |
|  | Extrahepatic cancer^a^ | 1.06 (0.65–1.73) | 0.805 |
|  | Alcohol use disorder | 0.78 (0.45–1.36) | 0.385 |
|  | Drug use disorder | 0.95 (0.46–1.95) | 0.894 |
|  | Depression/anxiety | 0.91 (0.54–1.54) | 0.736 |
| Results from a multivariable Cox regression model including all the variables in this table. Each individual was followed up from the date of resection until date of death, emigration, or 24 May 2024, whichever occurred first.  ALD: alcohol-related liver disease; CAD: coronary artery disease; CI: confidence interval; CKD: chronic kidney disease; COPD: chronic obstructive pulmonary disease; HBV: hepatitis B virus; HCV: hepatitis C virus; HR: hazard ratio; MASLD: metabolic dysfunction-associated steatotic liver disease; Rare liver diseases included primary sclerosing cholangitis, primary biliary cholangitis, and autoimmune hepatitis.  ^a^ Extrahepatic cancers diagnosed within 5 years prior to hepatocellular carcinoma diagnosis. | | | |

| Table S13. Factors associated with the likelihood of curative-intent treatment receipt among 1514 individuals with surveillance-detected hepatocellular carcinoma in Sweden, 2012–2022 (individual- and contextual-level socioeconomic status) | | | |
| --- | --- | --- | --- |
|  | | **Multivariable** | |
|  | | **aOR (95% CI)** | **P-value** |
| Sex | Male | 1.0 (ref) | - |
|  | Female | 0.96 (0.68–1.34) | 0.801 |
| Age group | 18–59 | 1.0 (ref) | - |
|  | 60–69 | 0.92 (0.63–1.37) | 0.694 |
|  | 70–79 | 0.55 (0.35–0.86) | 0.008 |
|  | 80+ | 0.32 (0.16–0.66) | 0.002 |
| Region of birth | Nordic | 1.0 (ref) | - |
|  | Europe (non-Nordic) | 1.08 (0.61–1.93) | 0.785 |
|  | Non-European | 0.94 (0.55–1.61) | 0.817 |
| Marital status | Married | 1.0 (ref) | - |
|  | Single | 0.95 (0.64–1.40) | 0.781 |
|  | Divorced | 1.00 (0.70–1.44) | 0.988 |
|  | Widowed | 0.84 (0.47–1.48) | 0.544 |
| Educational | >12 years | 1.0 (ref) | - |
| attainment | 10–12 years | 1.05 (0.72–1.53) | 0.808 |
|  | ≤9 years | 1.20 (0.80–1.81) | 0.373 |
| Household income | High | 1.0 (ref) | - |
|  | Medium | 0.56 (0.36–0.85) | 0.009 |
|  | Low | 0.46 (0.28–0.72) | 0.001 |
| Neighbourhood | Q1 (least) | 1.0 (ref) | - |
| deprivation | Q2 | 0.64 (0.37–1.08) | 0.096 |
|  | Q3 | 0.69 (0.42–1.16) | 0.162 |
|  | Q4 | 0.58 (0.36–1.04) | 0.099 |
|  | Q5 (most) | 0.68 (0.41–1.13) | 0.135 |
| Grade of urbanisation | Urban | 1.0 (ref) | - |
|  | Semi-urban | 1.11 (0.67–1.85) | 0.687 |
|  | Rural | 0.89 (0.60–1.31) | 0.562 |
| Period | 2012–2015 | 1.0 (ref) | - |
|  | 2016­–2019 | 0.90 (0.65–1.26) | 0.555 |
|  | 2020–2022 | 0.89 (0.61–1.28) | 0.518 |
| Aetiology | HCV | 1.0 (ref) | - |
|  | ALD | 0.88 (0.59–1.32) | 0.546 |
|  | HBV | 2.18 (1.30–3.65) | 0.003 |
|  | MASLD | 1.10 (0.65–1.86) | 0.725 |
|  | Rare liver diseases | 1.29 (0.74–2.24) | 0.371 |
|  | Cryptogenic | 0.98 (0.49–1.93) | 0.943 |
| CP score | ≤7 | 1.0 (ref) | - |
|  | >7 | 0.37 (0.25–0.55) | <0.001 |
| ECOG PS | 0–1 | 1.0 (ref) | - |
|  | ≥2 | 0.24 (0.17–0.34) | <0.001 |
| Tumour size | ≤20 mm | 1.0 (ref) | - |
| (largest) | 21–30 mm | 0.56 (0.38–0.81) | 0.002 |
|  | >30 mm | 0.13 (0.09–0.19) | <0.001 |
| N of tumours | 1 | 1.0 (ref) | - |
|  | 2–3 | 0.64 (0.48–0.86) | 0.003 |
|  | >3 | 0.14 (0.09–0.22) | <0.001 |
| Benign PVT |  | 0.18 (0.11–0.31) | <0.001 |
| Comorbidity | Type 2 diabetes | 1.05 (0.78–1.43) | 0.721 |
|  | CAD | 0.89 (0.60–1.32) | 0.551 |
|  | CKD | 1.34 (0.80–2.22) | 0.263 |
|  | COPD | 0.80 (0.54–1.19) | 0.266 |
|  | Extrahepatic cancer^a^ | 1.22 (0.81–1.81) | 0.341 |
|  | Alcohol use disorder | 1.05 (0.73–1.52) | 0.788 |
|  | Drug use disorder | 0.71 (0.42–1.22) | 0.216 |
|  | Depression/anxiety | 0.99 (0.65–1.52) | 0.979 |
| The multivariable logistic regression model, which included all the variables in this table, was statistically significant compared to the null model (Chi-square (42) = 584.75, p<0.001) and correctly classified 80% of cases.  ALD: alcohol-related liver disease; CAD: coronary artery disease; CI: confidence interval; CKD: chronic kidney disease; COPD: chronic obstructive pulmonary disease; CP: Child-Pugh; ECOG: Eastern Cooperative Oncology Group Performance Status; HBV: hepatitis B virus; HCV: hepatitis C virus; MASLD: metabolic dysfunction-associated steatotic liver disease; OR: odds ratio. PVT: benign portal vein thrombosis. Rare liver diseases included primary sclerosing cholangitis, primary biliary cholangitis, and autoimmune hepatitis.  ^a^ Extrahepatic cancers diagnosed within 5 years prior to hepatocellular carcinoma diagnosis. | | | |

| Table S14. Multivariable estimates for post curative-intent treatment mortality among 974 individuals with surveillance-detected hepatocellular carcinoma in Sweden, 2012–2022 (individual- and contextual-level socioeconomic status) | | | |
| --- | --- | --- | --- |
|  | | **Multivariable** | |
|  | | **aHR (95% CI)** | **P-value** |
| Sex | Male | 1.0 (ref) | - |
|  | Female | 1.07 (0.84–1.37) | 0.569 |
| Age group | 18–59 | 1.0 (ref) | - |
|  | 60–69 | 1.04 (0.78–1.38) | 0.794 |
|  | 70–79 | 1.39 (0.99–1.94) | 0.058 |
|  | 80+ | 2.27 (1.33–3.87) | 0.003 |
| Region of birth | Nordic | 1.0 (ref) | - |
|  | Europe (non-Nordic) | 0.60 (0.38–0.94) | 0.025 |
|  | Non-European | 0.97 (0.65–1.44) | 0.875 |
| Marital status | Married | 1.0 (ref) | - |
|  | Single | 0.95 (0.71–1.28) | 0.741 |
|  | Divorced | 1.07 (0.82–1.40) | 0.621 |
|  | Widowed | 0.93 (0.63–1.37) | 0.701 |
| Educational | >12 years | 1.0 (ref) | - |
| attainment | 10–12 years | 0.83 (0.63–1.10) | 0.197 |
|  | ≤9 years | 1.02 (0.77–1.37) | 0.881 |
| Household income | High | 1.0 (ref) | - |
|  | Medium | 1.03 (0.78–1.37) | 0.827 |
|  | Low | 1.43 (1.06–1.94) | 0.018 |
| Neighbourhood | Q1 (least) | 1.0 (ref) | - |
| deprivation | Q2 | 0.72 (0.50–1.03) | 0.072 |
|  | Q3 | 1.01 (0.73–1.41) | 0.941 |
|  | Q4 | 1.15 (0.82–1.62) | 0.419 |
|  | Q5 (most) | 0.98 (0.70–1.34) | 0.849 |
| Degree of urbanisation | Urban | 1.0 (ref) | 0.759 |
|  | Semi-urban | 1.35 (0.95–1.90) | 0.090 |
|  | Rural | 1.05 (0.78–1.39) | 0.763 |
| Period | 2012–2015 | 1.0 (ref) | - |
|  | 2016­–2019 | 0.75 (0.59–0.94) | 0.013 |
|  | 2020–2022 | 0.76 (0.56–1.04) | 0.088 |
| Aetiology | HCV | 1.0 (ref) | - |
|  | ALD | 1.54 (1.16–2.05) | 0.003 |
|  | HBV | 1.03 (0.72–1.46) | 0.886 |
|  | MASLD | 1.30 (0.90–1.88) | 0.165 |
|  | Rare liver diseases | 1.06 (0.69–1.62) | 0.790 |
|  | Cryptogenic | 1.67 (0.95–2.93) | 0.074 |
| Comorbidity | Type 2 diabetes | 1.04 (0.84–1.29) | 0.728 |
|  | CAD | 1.14 (0.86–1.52) | 0.363 |
|  | CKD | 1.73 (1.25–2.40) | 0.001 |
|  | COPD | 1.27 (0.95–1.70) | 0.101 |
|  | Extrahepatic cancer^a^ | 1.30 (0.99–1.70) | 0.055 |
|  | Alcohol use disorder | 1.14 (0.88–1.47) | 0.320 |
|  | Drug use disorder | 0.97 (0.65–1.43) | 0.867 |
|  | Depression/anxiety | 0.97 (0.73–1.30) | 0.861 |
| Treatment | Transplantation | 1.0 (ref) | - |
|  | Resection | 3.71 (2.49–5.52) | <0.001 |
|  | Ablation | 4.96 (3.40–7.23) | <0.001 |
| Results from a multivariable Cox regression model including all the variables in this table. Each individual was followed-up from the date of the first curative-intent treatment (transplantation, resection, or ablation) until date of death, emigration, or 24 May 2024, whichever occurred first.  ALD: alcohol-related liver disease; CAD: coronary artery disease; CI: confidence interval; CKD: chronic kidney disease; COPD: chronic obstructive pulmonary disease; HBV: hepatitis B virus; HCV: hepatitis C virus; HR: hazard ratio; MASLD: metabolic dysfunction-associated steatotic liver disease; Rare liver diseases included primary sclerosing cholangitis, primary biliary cholangitis, and autoimmune hepatitis.  ^a^ Extrahepatic cancers diagnosed within 5 years prior to hepatocellular carcinoma diagnosis. | | | |

**Figure S1. Proportions of stage at diagnosis and received treatment, stratified by sociodemographic characteristics and aetiology, among individuals aged ≥18 years with surveillance-detected hepatocellular carcinoma (HCC) in Sweden, 2012–2022.**

ALD: alcohol-related liver disease; HBV: hepatitis B virus; HCV: hepatitis C virus; MASLD: metabolic dysfunction-associated steatotic liver disease. Rare liver diseases included primary sclerosing cholangitis, primary biliary cholangitis, and autoimmune hepatitis.
